# Mitochondrial Metabolism Drives Low-density Lipoprotein-induced Breast Cancer Cell Migration

**DOI:** 10.1158/2767-9764.CRC-22-0394

**Published:** 2023-04-26

**Authors:** Sandrina Nóbrega-Pereira, Francisco Santos, Miguel Oliveira Santos, Teresa L. Serafim, Ana Patrícia Lopes, Diogo Coutinho, Filipa S. Carvalho, Rosário M. Domingues, Pedro Domingues, Bruno Bernardes de Jesus, Vanessa A. Morais, Sérgio Dias

**Affiliations:** 1 Instituto de Medicina Molecular João Lobo Antunes, Faculty of Medicine, University of Lisbon, Lisbon, Portugal.; 2 Instituto de Biomedicina (iBiMED), Department of Medical Sciences, University of Aveiro, Aveiro, Portugal.; 3 Mass Spectrometry Center, QOPNA, University of Aveiro, Aveiro, Portugal.; 4 Department of Chemistry and CESAM&ECOMARE, University of Aveiro, Aveiro, Portugal.

## Abstract

Most cancer-related deaths are due to metastases. Systemic factors, such as lipid-enriched environments [as low-density lipoprotein (LDL)-cholesterol], favor breast cancer, including triple-negative breast cancer (TNBC) metastasis formation. Mitochondria metabolism impacts TNBC invasive behavior but its involvement in a lipid-enriched setting is undisclosed. Here we show that LDL increases lipid droplets, induces CD36 and augments TNBC cells migration and invasion *in vivo* and *in vitro*. LDL induces higher mitochondrial mass and network spread in migrating cells, in an actin remodeling-dependent manner, and transcriptomic and energetic analyses revealed that LDL renders TNBC cells dependent on fatty acids (FA) usage for mitochondrial respiration. Indeed, engagement on FA transport into the mitochondria is required for LDL-induced migration and mitochondrial remodeling. Mechanistically, LDL treatment leads to mitochondrial long-chain fatty acid accumulation and increased reactive oxygen species (ROS) production. Importantly, CD36 or ROS blockade abolished LDL-induced cell migration and mitochondria metabolic adaptations. Our data suggest that LDL induces TNBC cells migration by reprogramming mitochondrial metabolism, revealing a new vulnerability in metastatic breast cancer.

**Significance:**

LDL induces breast cancer cell migration that relies on CD36 for mitochondrial metabolism and network remodeling, providing an antimetastatic metabolic strategy.

## Introduction

Cancer metabolism is a key aspect of tumor initiation and progression, and several metabolic adaptations carried by cancer cells to support tumor growth and proliferation have been described previously (1–3). Cancer cells are no longer thought to obtain their energy solely from glycolysis (the so-called “Warburg effect”), and, certainly, not because of mitochondrial defects (4, 5). Tumors are heterogeneous masses comprising different cell types, which can adapt to different metabolic programs and mitochondrial metabolism is increasingly recognized as an important axis in cancer progression (4–6). Apart from energy and anabolism, tricarboxylic acid cycle metabolites generated and metabolized in the mitochondria serve as substrates and cofactors for chromatin-modifying enzymes, thereby coupling mitochondrial metabolism and transcriptional regulation (7). Besides, additional layers of mitochondrial plasticity, including network dynamics and reactive oxygen species (ROS), are emerging as key determinants in cancer metastasis (8, 9).

There is compelling evidence, particularly in breast cancer, that invasive and metastatic cancer cells specifically favor mitochondrial respiration over aerobic glycolysis. It was shown that triple-negative breast cancer (TNBC) cells enhance oxidative phosphorylation (OXPHOS) and mitochondrial biogenesis during metastasis formation and rely on fatty acid oxidation (FAO) for the acquisition of an aggressive phenotype (10–13). Lipid metabolism is known to have a crucial role in cancer development and lipid synthesis and consumption in tumors have been under scrutiny (14). Vast epidemiologic literature suggests that obesity, and high cholesterol levels in particular, may result in greater tumor incidence and worse cancer patient outcomes (15–17). In particular, we have described that hypercholesterolemia in patient with untreated breast cancer strongly correlates with bigger and more metastatic tumors (18). Importantly, results from our lab and others have shown that lipid-enriched environments [particularly low-density lipoprotein (LDL)] favor tumor progression in experimental murine models of TNBC by modulating the tumor microenvironment and favoring aggressive cancer cell autonomous properties, including increased cell migration, proliferation, and metastatic behavior (19, 20).

Although a strong association between lipid-enriched environments and tumor progression has been postulated, the exact nature of the lipid-induced metabolic alterations that cell-autonomously fuel cancer cell aggressiveness is not well understood. Whether the acquisition of an invasive, prometastatic phenotype by breast cancer cells exposed to elevated systemic lipid levels—and of LDL in particular—is the result of specific mitochondria and metabolic alterations is undisclosed and was the focus of the current study.

## Materials and Methods

### Zebrafish Welfare and Handling

Zebrafish (*Danio rerio*) casper, nacre and Tg (*fli1:eGFP*) fish were handled and maintained according to standard protocols of the European Animal Welfare Legislation, Directive 2010/63/EU (European Commission, 2016) and Champalimaud Fish Platform. All protocols were approved by the Champalimaud Animal Ethical Committee and Portuguese institutional organizations—ORBEA (Orgão de Bem-Estar e Ética Animal/Animal Welfare and Ethics Body) and DGAV (Direção Geral de Alimentação e Veterinária/Directorate General for Food and Veterinary).

### Cell Culture

Human TNBC cells lines (MDA-MB-231, MDA-MB-436) were purchased from ATCC between 2012 and 2016, tested as being *Mycoplasma*-free and authenticated by examination of morphology and consistent *in vitro* performance. Cells were cultured in DMEM (Thermo Fisher Scientific) supplemented with 10% (v/v) heat-inactivated FBS (Gibco) and 1% Antibiotic-Antimycotic (Gibco) at 37°C and 5% CO_2_ atmosphere. Cell count ratio, proliferation, and wound-healing assays were performed according to ref. 19. For the wound-healing assay, Mitomycin C (Merck Milipore, 0.5 μmol/L) was used and images were acquired with 4× objective on a Zeiss Primovert microscope coupled with a Zeiss AxioCam ERc or an EVOS M500 microscope at time 0 hour and after 18/24 hours. Cells were treated with DMEM with 1% FBS-LPF (FBS-lipoprotein-free; Bio West) growth medium supplemented with LDL (Merck Milipore, 100 μg/mL), 2-Deoxi-D-glucose (2-DG) (Calbiochem, 2 mmol/L), oligomycin (Sigma, 2 μmol/L), etomoxir (Sigma, 100 μmol/L), sulfosuccinimidyl oleate (SSO; Sigma, 50 μmol/L), lysophosphatidic acid (LPA; Sigma, 10 μmol/L), *N*-acetylcysteine (NAC; Sigma, 5 mmol/L), MitoTEMPO (Sigma, 100 μmol/L), CK666 (Sigma, 50–75 μmol/L), anti-human LDL receptor blocking antibody (R&D Systems, 5 μg/mL), and DiI-LDL (Thermo Fisher Scientific, 100 μg/mL) for the indicated time. For palmitic acid (PA) and oleic acid (OA) treatment, sodium palmitate (P9767, Sigma-Aldrich) and sodium oleate (O7501, Sigma-Aldrich), respectively, were prepared according to ref. 21. Briefly, 2.5 mmol/L stock solution was prepared by dissolving in 1 mL solution of 0.1 mol/L NaOH and warming at 80°C until clear. The fatty acid (FA) solution was complexed with FA-free BSA (A7030, Sigma-Aldrich) in a molar ratio FA/BSA of 5:1, where 0.325 g BSA was dissolved in 8 mL of 0.9% NaCl, and the mixture was warmed to 45°C. The clear solution of FA solution was added drop-by-drop by pipet with agitation, filtered and stored at −20°C and cells were treated with 50–75 μmol/L PA or OA for the indicated times (21, 22). Lentiviral short hairpin RNA (shRNA) construct targeting *CD36* and a nontargeting scramble shRNA sequence (TRCN0000056999 Sigma; kindly provided by Dr. Salvador Aznar Benitah) were used as before (23). For shRNA transduction, lentiviruses were produced in HEK293T cells (CVCL_HA71) with covectors (pCMV delta R8.2, pMD2.G, Addgene_12263) using X-tremeGENE DNA Transfection Reagent (Roche), cells were transduced in the presence of 8 μg/mL polybrene and selected for 3 days with 2 μg/mL of puromycin (InvivoGen) in the culture media. For siRNA, cells were transfected with either control (nonspecific) single interference RNAs or directed against human CPT1A (EHU041711-20UG, Sigma) using Lipofectamine RNAiMAX transfection reagent (Invitrogen), with 6 hours between the two transfections (final concentration 10–30 nmol/L) following standard procedures, and cells were analyzed 48 hours after.

For zebrafish xenografts, MDA-MB-231 cells were treated with DMEM 1% FBS-LPF alone or supplemented with LDL for 48 hours. At day 3, cells were transfected with Mito-YFP (2–6 μg DNA) with FuGENE (Thermo Fisher Scientific) according to the manufacturer instructions. The next day, cells were detached with non-enzymatic methods (Cell Dissociation Buffer, enzyme-free, PBS; Thermo Fisher Scientific), washed with Dulbecco's PBS (DPBS) 1X (Bio West) and stained separately with the lipophilic dyes Vybrant CM-DiI (4 μL/mL in DPBS 1X) or Deep Red Cell Tracker (1 μL/mL in DPBS 1X, 10 mmol/L stock; Thermo Fisher Scientific), for 10 minutes at 37°C, in the dark.

### Zebrafish Xenografts

Fluorescently labeled control and LDL-treated cells were mixed (1:1 proportion at 0.5 × 10^6^ cells/μL) and injected under a fluorescence scope (Zeiss Axio Zoom V16) as in ref. 24. Approximately 500–1,000 cells were injected into the periviteline space (PVS) of 2 days post-fertilization (dpf) zebrafish embryo, previously anesthetized with Tricaine 1X (Sigma). Four days after injection, zebrafish xenografts were sacrificed, fixed with 4% (v/v) Formaldehyde (Thermo Fisher Scientific) at 4°C overnight and preserved at −20° C in 100% (v/v) methanol (MetOH).

### Immunofluorescence and Cell Staining

Zebrafish larvae whole-mount immunofluorescence was performed according to ref. 24. Briefly, xenografts were permeabilized with 0.1% (w/v) Triton X-100 (Sigma) in PBS 1x and blocked with a mixture of PBS 1X, 1% BSA, 1% DMSO (Sigma), 0.05% Triton X-100, 1.5% goat serum (Dako) for 1 hour at room temperature. Primary anti-GFP rabbit polyclonal antibody Alexa 488 conjugate (Invitrogen, 1:100) and DAPI (50 μg/mL; Merck Millipore) were used and xenografts were mounted with Mowiol (Sigma). Cells seeded in glass coverslips were fixed with 4% paraformaldehyde (Thermo Fisher Scientific) followed by permeabilization with 0.5% Triton X-100 and blocking with 0.5% BSA-PBS1x. For BODIPY 493/503 costaining, 0.2 mol/L glycine (Sigma), 0.1 mg/mL saponin (Sigma), and 3% BSA was used. Primary anti-human mouse HSP60 antibody (BD Biosciences, 1:250) or mouse TOM20 (F10, Santa Cruz Biotechnology, 1:200) was used followed by incubation with secondary antibodies donkey anti-mouse Alexa 488 (Invitrogen, 1:400), donkey anti-mouse Alexa 594 (Invitrogen; 1:500), or Alexa Fluor 594 Phalloidin (Thermo Fisher Scientific, 1:200), BODIPY 493/503 (Thermo Fisher Scientific; 0.2 μg/mL) and cells were mounted using Vectashield with DAPI (vectorlabs).

### Microscopy Acquisition and Analysis

For zebrafish xenografts imaging and analysis, initial screening was performed using an inverted fluorescence widefield microscope Zeiss Axiovert 200M, with 10× amplification. For the tropism evaluation, tiles of zebrafish larvae were acquired using the spinning disk confocal microscope Zeiss Cell Observer SD and quantified with ImageJ (SCR_003070, cell counter plugin). For the quantification of cells per organ, Imaris (SCR_007370) was used. For mitochondrial network quantification, confocal microscopy was performed in a Zeiss LSM 880 microscope and the images were acquired with a 63× oil objective (with 1.2–1.6× zoom). For live cell imaging, MitoTracker Deep Red (50 nmol/L) and BODIPY 493/503 (0.2 μg/mL; Thermo Fisher Scientific) were added in complete DMEM for 30 minutes and imaging was performed in a Zeiss Cell Observer Microscope with 63× oil objective. For DiI-LDL lipid area quantification, confocal microscopy was performed in a Zeiss LSM 710 microscope with 40× oil objective (with 1× zoom). Mitochondrial morphologic parameters, as number of mitochondria, mitochondria average area, mitochondria network (the area occupied by mitochondria particles by the cell tracer area or phalloidin staining area), mean elongation and DiI-LDL lipid area were quantified using Fiji (SCR_002285) and the “Analyze particles” tool.

### Flow Cytometry

Cell staining and acquisition was performed as in ref. 3. Briefly, live cells were incubated with BODIPY 493/503 (Thermo Fisher Scientific, 0.2 μg/mL) for 10 minutes at room temperature, MitoTracker Deep Red (Thermo Fisher Scientific, 2 nmol/L) for 15 minutes at room temperature or CellROX (Thermo Fisher Scientific, 2 μmol/L) for 20 minutes at 37°C. For CD36 cell surface staining, cells were blocked with PBS-0.5% BSA, incubated with anti-human mouse CD36 (BioLegend; 1:100) followed by secondary antibody goat anti-mouse Alexa 633 (Invitrogen, 1:400). Analysis was performed using LSR Fortessa or BD Accuri (BD Biosciences) flow cytometers and FlowJo (SCR_008520, LLC).

### qPCR

For RNA analysis, total RNA from cells was extracted using TRIzol (Life Technologies) and samples were reverse transcribed using random priming and Superscript Reverse Transcriptase (Life Technologies), according to the manufacturer's instructions. qPCR was performed using DNA master SYBR Green I mix (Applied Biosystems) in an ABI PRISM 7700 thermocycler and applying the 2^(−ΔΔ*C*t)^ method. mitochondrial DNA (mtDNA) determination was performed as in refs. 3, 25 using phenol:chloroform:isoamyl alcohol (Sigma) extraction and human mitochondrial ND1 (mtND1) relative to nuclear β2-microglobulin gene determination. All primer sequences are described in Supplementary Table S1.

### Transmission Electron Microscopy

Cells were processed and acquired as in ref. 3. Cells were fixed with 2.5% glutaraldehyde (Electron Microscopy Sciences) and 0.1% formaldehyde (Thermo Fisher Scientific) in 0.1 mol/L cacodylate buffer (Sigma) at pH7.3 and treated with 0.1% Millipore filtered cacodylate buffered (Sigma), followed by postfixation with 1% Millipore-filtered osmium tetroxide (Electron Microscopy Sciences) and staining with 1% Millipore-filtered uranyl acetate (Agar Scientifics) and lead citrate (Sigma). Sections were cut in a Reichert supernova microtome and examined in an H-7650 transmission electron microscope (Hitachi) at an accelerating voltage of 100 kV. Digital images were obtained using a XR41M mid mount AMT digital camera (Advanced Microscopy Techniques Corp). For quantification, the number of mitochondria per cell was counted in 3,000× magnification sectioned images.

### Western Blots

Whole-cell extracts were prepared using RIPA buffer containing proteinase inhibitors (Roche) and resolved using SDS-PAGE (Mini-PROTEAN TGX, Bio-Rad) under reducing conditions (β-mercaptoethanol, Bio-Rad) and Precision Plus Protein Standards (dual color, Bio-Rad) was used. Proteins were subsequently blotted onto a nitrocellulose membrane (Bio-Rad) and hybridized using anti-human antibodies against β-ACTIN (Sigma, 1:15,000), DNM1L (Santa Cruz Biotechnology, 1:200), MFN1 and MFN2 (Abcam, 1:1,000), PPARGC1A (Calbiochem, 1:1,000), and horseradish peroxidase–coupled secondary antibodies (1:6,000, Promega). The Pierce enhanced chemiluminescence (Thermo Fisher Scientific) detection system and Fuji Medical X-ray Films (FUJIFILM) was used. Quantification of densitometric units was performed using Scion Image (SCR_008673).

### Seahorse Energetic Assays

MDA-MB-231 cells were seeded into the specialized XF24 cell culture microplate (Seahorse Bioscience), at 24,000 cells/well and maintained in DMEM 1% FBS-LPF (control) or LDL-exposed cells in the presence or absence of SSO (Sigma, 50 μmol/L) for 36 hours. Measurements were performed in a Seahorse Bioscience Extracellular Flux Analyzer (Agilent).

For the Seahorse Cell Mito Stress Test profile, cells were incubated for 1 hour at 37°C in XF Seahorse medium supplemented with glucose, glutamine, and sodium pyruvate and without CO_2_. Oxygen consumption rate (OCR) was measured under standard conditions and after the addition of 1 μmol/L oligomycin, 1.5 μmol/L FCCP, and 1 μmol/L rotenone/1 μmol/L Antimycin A. Extracellular acidification rate (ECAR) was measured under standard conditions and after the addition of 10 mmol/L glucose, 1 μmol/L oligomycin, and 20 mmol/L 2-DG. Real-time measurements (quintuplicates) of the OCR (picomoles per minute) and ECAR (mpH per minute) were plotted over time. Glycolysis, non-glycolysis, glycolytic capacity, and glycolytic reserve were calculated as follows: Glycolysis = (maximum rate measurement before oligomycin injection) − (final rate measurement before 2-DG injection); Non-glycolysis = Last rate measurement prior to glucose injection; Glycolytic capacity = (maximum rate measurement after oligomycin injection) − (final rate measurement before glucose injection); and Glycolytic reserve = (glycolytic capacity) − (glycolysis).

For the Seahorse Mito Fuel Flex Test, cells were switched to XF Seahorse medium containing glucose and glutamine, and were incubated for 1 hour at 37°C in CO_2_ privation, prior to the assay. Glucose dependency and capacity assays were measured under standard conditions and after the addition of 6 μmol/L UK5099, followed by the addition of 8 μmol/L etomoxir/4 μmol/L BPTES and 1 μmol/L rotenone/1 μmol/L Antimycin A; and the FAO dependency and capacity assays were measured after addition of 8 μmol/L etomoxir, followed by the addition of 6 μmol/L UK5099/4 μmol/L BPTES and 1 μmol/L rotenone/1 μmol/L Antimycin A. Fuel dependency indicates the measurement of cells’ reliance on a particular fuel pathway to maintain baseline respiration [Dependency (%) = [(baseline OCR − Target 1 inhibitor OCR)/(Baseline OCR − All three inhibitor OCR)] × 100). Fuel capacity indicates the ability of a cell's mitochondria to oxidize a fuel when other fuel pathways are inhibited (Capacity (%) = [(baseline OCR − Other two inhibitor's OCR)/(Baseline OCR − All three inhibitor OCR)] × 100). Fuel flexibility indicates the difference between fuel capacity and dependency, which is the ability of cells to increase oxidation of a particular fuel to compensate for inhibition of alternative fuel pathway(s) [Flexibility (%) = Capacity (%)− Dependency (%)].

Cells were then lysed in RIPA buffer and subjected to Pierce BCA protein assay (Thermo Fisher Scientific). OCR and ECAR values were normalized by protein values in each well.

### Mitochondrial Isolation

MDA-MB-231 cells were treated with DMEM 1% FBS-LPF (control) or LDL-exposed cells in the presence or absence of etomoxir (Sigma, 100 μmol/L) for 24 hours. Isolation of mitochondria from the cell pellet was performed at 4°C according to refs. 26, 27. Briefly, the cell pellet was suspended in isolation buffer (250 mmol/L sucrose, 1 mmol/L ethylene glycol-bis(β-aminoethyl ether)-N,N,Nʹ,Nʹ-tetraacetic acid (EGTA), 10 mmol/L HEPES, and 5 g/L BSA pH 7.5) and centrifuged at 500 × *g* for 2 minutes. The supernatant was discarded and the remaining pellet was suspended in isolation buffer. The cell suspension was homogenized in a tight-fitting Potter homogenizer (Teflon pestle). The supernatant was centrifuged at 10,000 × *g* for 10 minutes. The mitochondrial pellet was washed with BSA-free isolation buffer and protein content was determined with Bio-Rad RC-DC assay.

### Extraction of Mitochondrial Phospholipids and Quantification by Phosphorus Assay

Lipid extraction of mitochondrial fraction was performed according to the Bligh and Dyer method as before (27, 28). Briefly, 3.75 mL of chloroform/methanol 1:2 (v/v) was added to 1 mL of mitochondrial fraction. The tubes were well vortexed and an additional volume of 1.25 mL chloroform and 1.25 mL milli-Q H_2_O were added. Following vigorous vortex, samples were centrifuged at 2,000 rpm (Mixtasel centrifuge, Selecta), the organic phase was collected to a new glass tube and the remaining biomass residue was reextracted by adding 1.88 mL of chloroform. The lipid extracts were dried in a nitrogen flow and stored at −20°C. The amount of total phospholipid (PL) in lipid extracts was determined using the phosphorous assay as before (27). The dried samples were resuspended in 80 μL of dichloromethane, of which an aliquot of 10 μL was dried and incubated 1 hour at 180°C with 125 μL of perchloric acid (70%) in a heating block (Stuart) Once cooled, the solutions were mixed by vortexing with 825 μL of Milli-Q water, 125 μL of (NH_4_)_6_Mo_7_O_24·4_H_2_O) 2.5% and 125 μL of ascorbic acid 10% freshly prepared. Samples and standards (8 standard solutions with 0.1–2 μg of phosphorus) were simultaneously incubated at 100 °C in a water bath for 10 minutes and cooled down. The content of inorganic acid was measured in a microplate spectrophotometer (Multiscan 90, Thermo Fisher Scientific) at 797 nm.

### FA Derivation and Analysis by Gas Chromatography–Mass Spectrometry

For FA derivatization, 10 μg of previously dried PL extracts were resuspended in the corresponding volumes of chloroform and dried under a nitrogen flow, followed by the addition of 1 mL of C19:0 internal standard solution in hexane (0.99 μg/mL, methyl nonadecanoate, 74208, Sigma). Then, 200 μL of KOH (2 mol/L) was added to each sample, followed by vortex, addition of 2 mL of NaCl and centrifugation at 2,000 rpm for 5 minutes (Pro-Analytical series). Then, 600 μL of the upper phase was dried under a nitrogen stream and 100 μL of hexane was added. Gas chromatography–mass spectrometry (GC-MS) analysis was carried out by injection of 2 μL solution of FAME mixture in hexane, obtained after the derivatization, in the Agilent 8860 GC System gas chromatograph with GC 5977B Network Mass Selective Detector, operating at 70 eV at 250°C, and equipped with a DBFFAP (Agilent 123-3232. 30 m × 320 μm × 0.25 μm) column. The FA methyl esters were identified using Agilent MassHunter Qualitative analysis (SCR_019081), supported by NIST2014 mass spectral library, by comparing their retention time and mass spectrometry (MS) spectra with those of Sigma-Aldrich standards (37 Component FAME Mix, Sigma), and by MS spectra comparison with online databases (AOCS lipid library). Integration and quantitative analysis of FAs was performed from calibration curves of each FA methyl ester from a FAME mixture (Supelco 37 Component FAME Mix, CRM47885, Sigma), analyzed by GC-MS under the same conditions of extracts. The relative amounts of FAs were calculated by the ratio of the amount of each FAME and the sum of all identified FAMEs and the results expressed as means (%).

### Statistical Analysis

Zebrafish xenografts tropism and mitochondrial network statistical analyses were performed using Fisher exact test and *χ*^2^, whereas the remaining was performed using two-tailed Student *t* test and one-way or two-way ANOVA followed by Tukey post-test for multiple comparisons with GraphPad Prism (SCR_002798). Pathway analysis of transcriptomes from microarrays data (19) was performed using the DAVID Functional Annotation tool (29, 30) and applying a statistic cutoff [−*log10 (0.05)*] for Benjamini FDR correction. For FA profile by GC-MS, multivariate or univariate analysis was performed using R version 3.5 in Rstudio version 1.1.4., and a level of 0.95. Kruskal–Wallis test followed by Tukey or Dunn *post hoc* comparisons were performed with the R built-in function. Heatmaps were created using the R package pheatmap using “Euclidean” as clustering distance, and “ward.D” as the clustering method. Data are presented as mean ± SD. *, *P* < 0.05; **, *P* < 0.01; ***, *P* < 0.001; ****, *P* < 0.0001.

### Data Availability

The data generated in this study are available within the article, its Supplementary Data and upon request to the corresponding author.

## Results

### LDL-exposed Breast Cancer Cells Display Enhanced Invasiveness and Differential Mitochondrial Network in Xenotransplanted Zebrafish Larvae

To explore the impact of LDL exposure on the behavior of breast cancer cells *in vivo*, we xenotransplanted human TNBC MDA-MB-231 cells into zebrafish larvae as a model to study invasion and metastatic spread (24). In particular, we investigated whether preexposure of MDA-MB-231 cells to LDL (2 days) would impact their migratory and invasive behavior *in vivo* and whether this was accompanied by alterations in the mitochondria network. As both conditions were inoculated in the same larvae, we used different cell tracers (DiI for control and Cy5 for LDL-exposed cells) to discriminate the experimental groups and cells were additionally transiently transfected with the mitochondrial reporter Mito-YFP (31).

**FIGURE 1:**
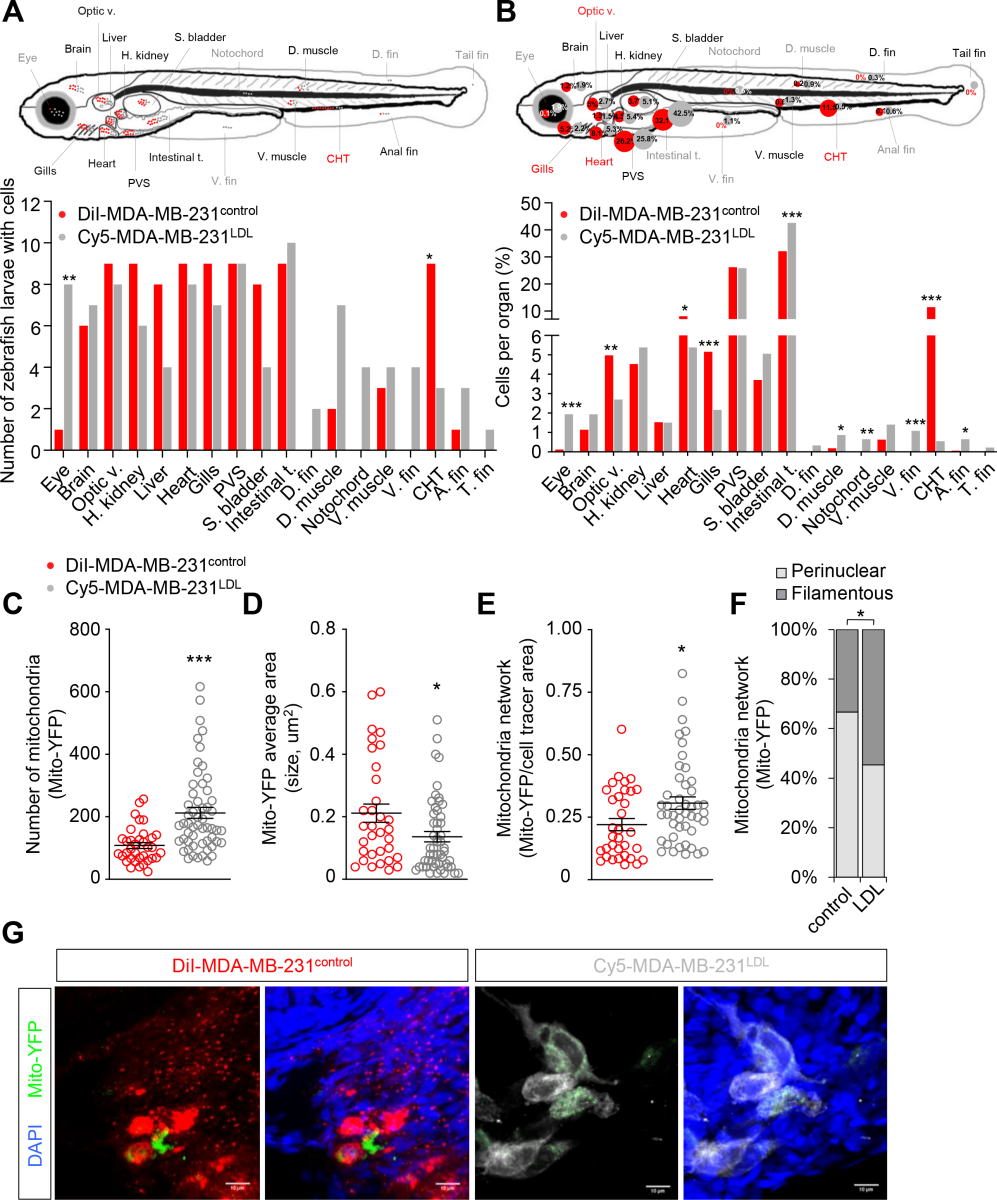
LDL-exposed TNBC cells show different invasion potential and mitochondrial network distribution in xenotransplanted zebrafish larvae at 4 dpi (6 dpf). **A,** Top, Schematic representation of MDA-MB-231 cell invasion potential throughout the zebrafish body in xenotransplanted larvae at 2 dpf and analyzed at 4 dpi (6 dpf) with DiI-labeled MDA-MB-231 control (red) and Cy5-labeled LDL-exposed (gray) cells detected in the indicated organs (gray and red depict organs with increase tropism for LDL-exposed and control cells, respectively, and each dot represents one xenograft). Bottom, Quantification of the invasive potential depicted as the number of zebrafish larvae xenografts (*n* = 11 from one independent experiment) with DiI-labeled MDA-MB-231 control (red) and Cy5-labeled LDL-exposed (gray) cells in the indicated organs. **B,** Top, Schematic representation of the total distribution of labeled MDA-MB-231 control (red, *n* = 1567) and Cy5-labelled LDL-exposed (gray, *n* = 931) cells throughout the zebrafish larvae (*n* = 11), each circle represents the proportion of cells of each condition in that organ (gray and red depict organs with statistical significance for LDL-exposed and control cells, respectively). Bottom, Quantification of cells distribution depicted as the percentage of DiI-labeled MDA-MB-231 control (red) and Cy5-labeled LDL-exposed (gray) cells present in the indicated organs over total. Total Mito-YFP number (**C**), average area (**D**), and mitochondria network (**E**) determined as the Mito-YFP area by cell tracer DiI or Cy5 area (for control and LDL cells, respectively) in control (*n* = 33/35) and LDL-exposed (*n* = 46/55) MDA-MB-231 cells throughout the body of immunolabeled xenotransplanted zebrafish larvae (*n* = 11). **F,** Chart representing Mito-YFP-labeled mitochondrial network distribution of control (*n* = 39) and LDL-exposed (*n* = 64) MDA-MB-231 cells in the xenotransplated zebrafish larvae. **G,** Representative images of maximum intensity projection of DiI-labelled MDA-MB-231 control (red) and a Cy5-labeled MDA-MB-231LDL (gray) cells expressing Mito-YFP (green) and displaying a perinuclear (control) and filamentous (LDL) mitochondrial network distribution acquired from the PVS (control) and eye (LDL) of xenotransplanted zebrafish larvae at 4 dpi. Nuclei are labeled with DAPI (blue). Scale bar, 10 μm (Micrometer). A., anal; CHT, caudal hematopoietic tissue; D., dorsal; H. kidney, head kidney; Intestinal t., intestinal tract; Optic v., optic vesicle; PVS, perivitelline space; S. Bladder, swim bladder; T., tail; V., ventral. Data are presented as mean ± SD. Each circle in the plot **C**–**E** represents individual cell measurement. *, *P* < 0.05; **, *P* < 0.01; ***, *P* < 0.001.

We first analyzed the ability of LDL-exposed and control MDA-MB-231 cells to migrate to distant organs in the zebrafish larvae at 4 days post-injection (dpi; corresponding to 6 dpf) using inverted spinning disk confocal microscopy (Fig. 1A). LDL-exposed MDA-MB-231 cells presented a trend toward a differential migration pattern in the zebrafish larvae as compared with control cells (*χ*^2^*P* = 0.06, *n* = 11; Fig. 1A; Supplementary Fig. S1A), with a significant number of zebrafish larvae accumulating LDL-exposed cells in the eye and control cells in the caudal hematopoietic tissue (CHT), a highly vascularized region with a dense plexus unifying caudal artery and caudal vein (32). Moreover, there was an increased number of zebrafish larvae displaying LDL-exposed MDA-MB-231 cells at distant sites, being the dorsal fin, notochord, ventral fin and tail fin invaded solely by LDL-exposed MDA-MB-231 cells (Fig. 1A).

To assess differential cell distribution in the whole organism, we quantified the number of individual cells in each organ and the presence of tumor cell masses in xenotransplanted zebrafish larvae at 4 dpi. Similarly, LDL-exposed MDA-MB-231 cells were found in all organs analyzed (Fig. 1B). Notably, less vascularized organs were invaded preferentially by LDL-exposed MDA-MB-231 cells (Fig. 1B). Of these, some were pierced by collateral vessels (dorsal muscle and ventral muscle), by a single vessel (eye), or lied just beneath CHT (anal fin) and were still invaded by MDA-MB-231 control cells; others (such as notochord, ventral fin, dorsal fin, tail fin) had no detectable vessels in angiographic studies (32) and in xenotransplanted Tg (*fli1:eGFP*) zebrafish larvae at 6 dpf (Supplementary Fig. S1B) and were exclusively infiltrated by LDL-exposed MDA-MB-231 cells (Fig. 1B). Although MDA-MB-231 control cells were found in higher proportion in extremely vascularized organs (such as optic vesicle, heart, gills, and CHT), LDL-exposed MDA-MB-231 cells could also invade those vascularized organs displaying for instance preferred accumulation in the intestinal tract (Fig. 1B).

Next, we assessed whether LDL-exposed TNBC cells present mitochondrial alterations in the xenotransplanted zebrafish larvae, using the Mito-YFP reporter. We observed an overall increased number of Mito-YFP particles in zebrafish larvae xenotransplanted with LDL-exposed MDA-MB-231 cells (Fig. 1C). This difference was statistically significant in most organs analyzed, including PVS, intestinal tract, and brain (Supplementary Fig. S1C). Moreover, the average area of Mito-YFP particles, which reflects the size of mitochondria, was overall reduced in LDL-exposed MDA-MB-231 cells xenotransplanted into zebrafish larvae (Fig. 1D) and this difference was particularly prominent in the PVS and intestinal tract (Supplementary Fig. S1D). Finally, we quantified the mitochondrial network as the total area occupied by Mito-YFP particles in the cell area delineated by the DiI and Cy5 tracers and in general, LDL-exposed MDA-MB-231 cells presented a higher mitochondrial network in xenotransplated zebrafish larvae as compared with control MDA-MB-231 cells (Fig. 1E) and this difference was significant in the brain (Supplementary Fig. S1E). We could observe two phenotypes of mitochondrial network distribution: mitochondrial aggregates (clusters) around the nucleus, often located unilaterally, which we designated perinuclear mitochondria (Fig. 1F and G) and long mitochondrial filaments, located unilaterally or bilaterally around the nucleus and distributed throughout the whole cell, designated as filamentous mitochondria (Fig. 1F and G). The majority of LDL-exposed MDA-MB-231 migrating cells in zebrafish larvae presented a filamentous distribution (54.7% vs. 33.3% for control cells) contrasting with control cells that adopted preferentially a perinuclear mitochondrial network (66.7% vs. 45.3% for LDL cells). Filamentous distribution was more prevalent in LDL-exposed MDA-MB-231 cells in most organs analyzed, being particularly evident in the brain, in agreement with the qualitative analysis (Supplementary Fig. S1F). These results suggest that LDL-exposure cell-autonomously induce invasive behavior and mitochondrial remodeling of MDA-MB-231 cells in xenotransplated zebrafish larvae.

### LDL Exposure Induces Mitochondrial Network Spread Distribution and Destabilized Cristae in Migrating Breast Cancer Cells

**FIGURE 2:**
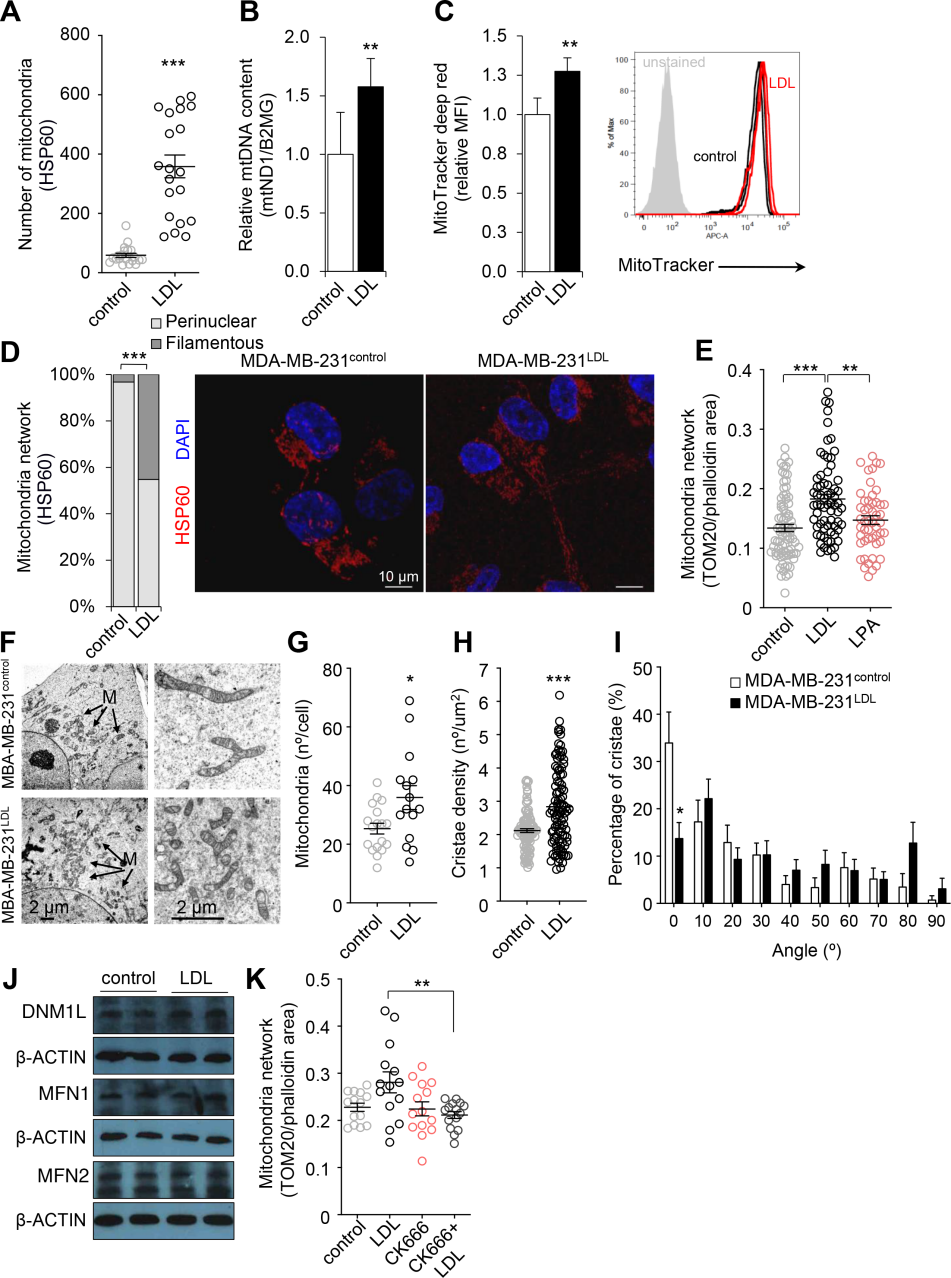
LDL-exposed migrating TNBC cells display differential mitochondrial network and cristae destabilization. **A,** Total HSP60–labeled mitochondria number in control and LDL-exposed migrating MDA-MB-231 cells (*n* = 20 each). **B,** mtDNA content accessed by qPCR analysis of the human mitochondrial ND1 gene relative to the nuclear β2-microglobulin gene in DNA samples from untreated (control) or LDL-exposed MDA-MB-231 cells (*n* = 7 each). **C,** Flow cytometry quantification of MitoTracker Deep Red staining depicted as relative median fluorescence intensity (MFI; left) in untreated (control) or LDL-exposed MDA-MB-231 cells and histograms (right; *n* = 5 each). **D,** HSP60–labeled mitochondrial network distribution of control and LDL-exposed migrating MDA-MB-231 cells (left; *n* = 31 each) and representative images of cells displaying perinuclear (control) and filamentous (LDL) mitochondrial network distribution (right; 63× objective). Nuclei are labeled with DAPI (blue). Scale bar, 10 μm (Micrometer). **E,** Mitochondrial network determined as the TOM20 total area over the phalloidin area of immunolabeled control, LDL- or LPA-exposed MDA-MB-231 migrating cells (*n* = 50/82 cells per condition). Representative TEM images (left 1,500× and right 3,000×; Scale bar, 2 μm (Micrometer); M: mitochondria; **F**) and quantitative plots of mitochondria number per cell (*n* = 15/18 cells; **G**), cristae density as the number of cristae per μm^2^ mitochondrial area (*n* = 100 mitochondria; **H**) and percentage of cristae in the angles displayed (*n* = 12/27 mitochondria; **I**) in untreated (control) or LDL-exposed MDA-MB-231 cells. **J,** Western blot analysis for DNM1L, MFN1, and MFN2 proteins in untreated (control) or LDL-exposed MDA-MB-231 cells (representative images, uncropped images of blots are shown in Supplementary Fig. S8). **K,** Mitochondrial network determined as the TOM20 total area over the phalloidin area of immunolabeled control or LDL-exposed MDA-MB-231 migrating cells in the absence or presence of CK666 (*n* = 10/15 cells per condition). Data are presented as mean ± SD. Each circle in the plot **A**, **E**, **G**, **H**, and **K** represents individual cell measurement. *, *P* < 0.05; **, *P* < 0.01; ***, *P* < 0.001.

To explore the mechanistic action of LDL, we characterized the mitochondrial alterations upon LDL exposure in migrating MDA-MB-231 cells *in vitro*. We previously reported that LDL-exposed MDA-MB-231 cells proliferate two times more and display increased migratory capacity in a wound-healing assay (19). In accordance with zebrafish xenotransplanted larvae, MDA-MB-231 migrating cells in the wound-healing assay (with Mitomycin C for cell-cycle arrest) exhibit increased number of heat shock protein (HSP60)–labeled mitochondria in the presence of LDL (Fig. 2A). In addition, we observed increased mitochondrial mass as determined by mtDNA content and MitoTracker Deep Red flow cytometry staining in LDL-exposed MDA-MB-231 migrating cells (Fig. 2B and C). LDL exposure promoted a similar effect in the alternative TNBC cell line MDA-MB-436, with increased mitochondrial mass and migration in the wound-healing assay (Supplementary Fig. S2A and S2B). Moreover, in agreement with the *in vivo* data, the impact of LDL exposure in the HSP60–labeled mitochondrial network of MDA-MB-231 migrating cells was very striking (Fig. 2D) with virtually all control cells presenting a perinuclear (around the nucleus) mitochondrial network (96.8% vs. 54.8% for LDL cells) whereas the majority of LDL-exposed cells exhibited a filamentous (extended through the cell area) network distribution (45.2% vs. 3.2% for control cells). To account for technical artefacts inherent to cell fixation, we confirmed these results using live MitoTracker Deep Red imaging (Supplementary Fig. S2C). To investigate whether mitochondrial network adaptations in migrating MDA-MB-231 cells was exclusive to LDL exposure or more broadly applied to other agents that induce cell migration, we treated cells with LPA, a PL ligand that stimulates cell migration through activation of PI3K/PAK1/ERK signaling (33). As for LDL, MDA-MB-231 cells exposed to LPA present increased migration in the wound-healing assay (Supplementary Fig. S2D). However, and as opposed to LDL, LPA exposure did not significantly alter the number of translocase of outer mitochondrial membrane 20 (TOM20) mitochondrial particles (Supplementary Fig. S2E) or the mitochondrial network, quantified as the area occupied by TOM20 mitochondrial particles in the total cell area (delineated by phalloidin staining; Fig. 2E).

We used transmission electron microscopy (TEM) to obtain high resolution micrographs and evaluate the organization of the mitochondrial inner membrane cristae. TEM analysis confirmed that LDL-treated MDA-MB-231 cells present increased number of mitochondria (Fig. 2F and G) and a tendency for decreased average area of mitochondrial particles (Supplementary Fig. S2F), as observed in the xenotransplanted zebrafish larvae (Fig. 1D). LDL-exposed cells presented mitochondria with more heterogeneous cristae density compared with control cells that display cristae number ranging from 1 to 4 cristae per μm^2^ (Fig. 2H). Moreover, the mitochondria of LDL cells presented decreased proportion of cristae with smallest incident angle (0°; Fig. 2I), reflecting fewer parallel cristae arrangement. Mitochondria are highly dynamic organelles that undergo continuous changes in morphology, degree of biogenesis, and mitophagy (34). The expression of several mitochondrial biogenesis factors, including the master regulator PPARG coactivator 1 alpha *(PPARGC1A)* and the FAO coactivator peroxisome proliferator activated receptor alpha *(PPARA)* were either unchanged or reduced in migrating MDA-MB-231 cells exposed to LDL (Supplementary Fig. S2G). Regarding mitochondrial dynamics, LDL-exposed MDA-MB-231 cells display increased expression of the mitochondrial fission protein dynamin 1 like (DNM1L) with no difference in the expression of the mitochondrial fusion proteins mitofusin 1 and 2 (MFN1 and MFN2) (Fig. 2J; Supplementary Fig. S2H). Quantification of mitochondrial morphology (35) revealed that MDA-MB-231 cells present an elongation score close to 1 (Supplementary Fig. S2I) under control or LDL exposure, indicative of fragmented mitochondria. Cell migration is a highly energetic process that requires a dynamic remodeling between mitochondria energetic processes and actin cytoskeleton as mitochondria deliver localized ATP for actin related protein 2/3 complex (Arp2/3) network growth during cell invasion (36). Indeed, cotreatment with the Arp2/3 inhibitor CK666 (37) abolished the LDL-induced migratory effect (Supplementary Fig. S2J), and the LDL-induced increase network distribution in migrating MDA-MB-231 cells (Fig. 2K), highlighting that LDL-induced migration and mitochondrial network spread requires actin cytoskeleton remodeling.

### LDL-induced Breast Cancer Cell Migration is Mediated by the FA Transporter CD36

**FIGURE 3:**
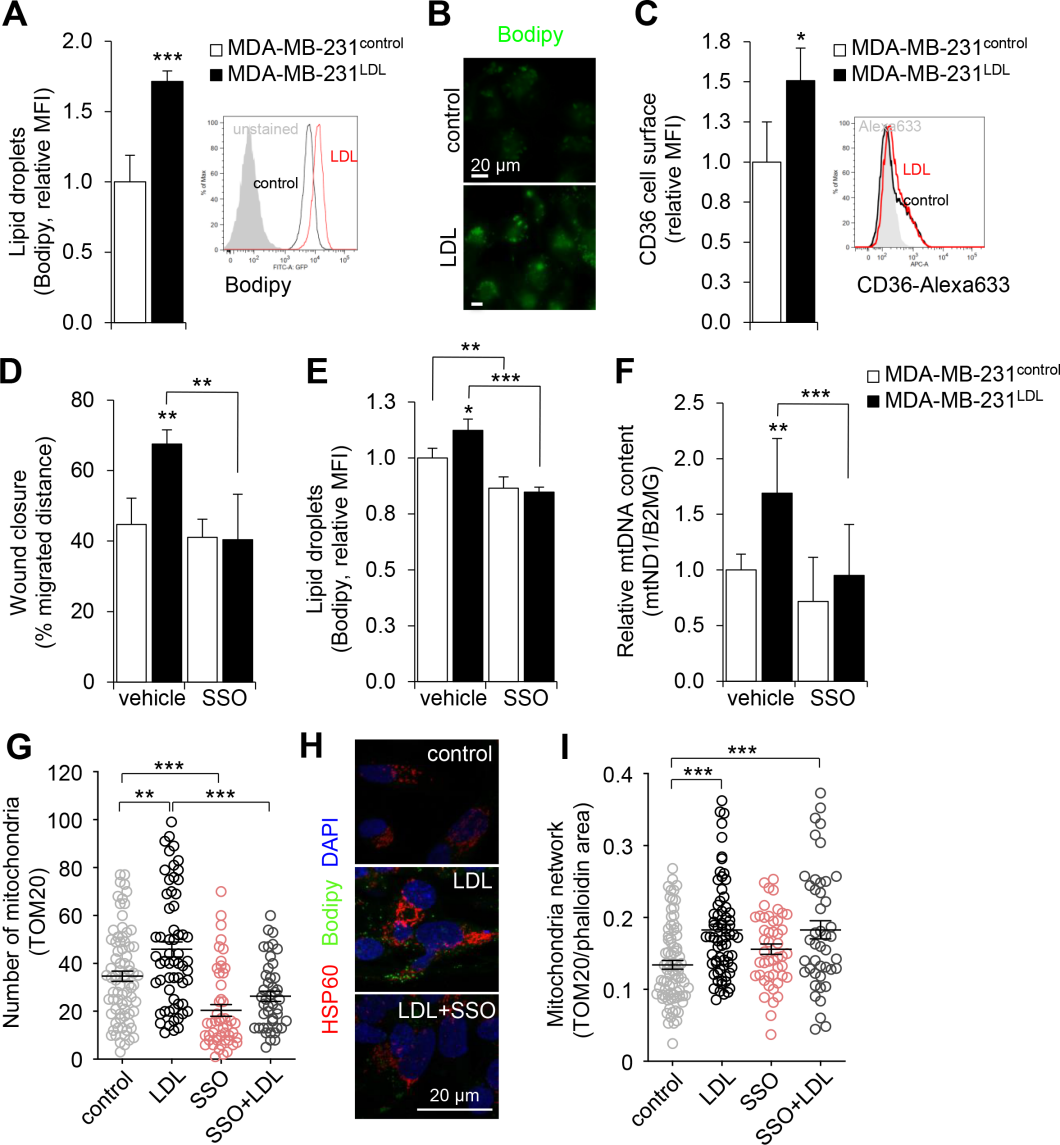
LDL-induced breast cancer cell migration is mediated by the FA transporter CD36. **A,** Flow cytometry quantification of BODIPY 493/503 (Bodipy) staining depicted as relative median fluorescence intensity (MFI; left) in untreated (control) or LDL-exposed MDA-MB-231 cells (*n* = 5 each) and representative histograms (right). **B,** Representative live fluorescent microscopy images of control and LDL-treated MDA-MB-231 cells stained with BODIPY 493/503 (Bodipy, 63× objective; scale bar, 20 μm (Micrometer). **C,** Flow cytometry quantification of CD36 cell surface expression depicted as fold MFI (left) in untreated (control) or LDL-exposed MDA-MB-231 cells and histograms (right; *n* = 4 each). **D,** Migratory capacity represented as percentage of wound closure of control or LDL-exposed MDA-MB-231 cells in the absence (vehicle) or presence of SSO (50 μmol/L; *n* = 4 each). **E,** Flow cytometry quantification of BODIPY 493/503 (Bodipy) staining of control or LDL-exposed MDA-MB-231 cells in the absence (vehicle) or presence of SSO (*n* = 4 each). **F,** Relative mtDNA content from control or LDL-exposed MDA-MB-231 cells in the absence (control) or presence of SSO (*n* = 6/8 each). **G,** Number of TOM20–labeled mitochondria in control or LDL-exposed migrating MDA-MB-231 cells in the absence (vehicle) or presence of SSO (*n* = 44/78 cells per condition). **H,** Representative images of control, LDL and LDL+SSO treated MDA-MB-231 migrating cells immunolabeled for HSP60 (red) and lipid droplets (Bodipy, green). Nuclei are labeled with DAPI (blue). 63× objective; scale bar, 20 μm (Micrometer). **I,** Mitochondrial network determined as the TOM20 total area over the phalloidin area of immunolabeled control or LDL-exposed MDA-MB-231 migrating cells in the absence or presence of SSO (*n* = 44/82 cells per condition). Data are presented as mean ± SD. Each circle in the plot **G** and **I** represents individual cell measurement. *, *P* < 0.05; **, *P* < 0.01; ***, *P* < 0.001.

Lipoproteins are complex particles composed of multiple proteins and lipids, including cholesterol, PLs, and triglycerides. Once internalized, LDL is hydrolyzed in the endosome and lysosome and lipids are stored into lipid droplets, being subsequently mobilized for several cellular processes, including ATP production through FAO (38). Indeed, we observed that exposure of MDA-MB-231 cells to LDL for 48 hours resulted in a significant increase in lipid droplets as assayed by the neutral lipid dye BODIPY 493/503 by flow cytometry and live imaging (Fig. 3A and B). Several types of membrane LDL receptors and scavengers mediate the endocytosis of native and modified LDL, including the LDL receptor (LDL-R), sterol regulatory element binding transcription factor 1 (SREBP1) and CD36 (39, 40). We observed that 48 hours exposure to LDL promoted a dramatic reduction in the expression of *LDL-R* and *SREBP1* in MDA-MB-231 cells (Supplementary Fig. S3A) whereas the expression of *CD36* transcript and cell surface protein is augmented (Fig. 3C; Supplementary Fig. S3A). To explore the requirement of CD36 for LDL-induced TNBC cell migration, we inhibited CD36 using the small-molecule inhibitor SSO (41) and observed that it abrogated the LDL-induced migration of MDA-MB-231 cells in the wound-healing assay and the increase in lipid droplets content (Fig. 3D and E). Similarly, shRNA-mediated depletion of CD36 (Supplementary Fig. S3B) abolished the LDL-induced migration of MDA-MB-231 cells and the increase in lipid droplet content (Supplementary Fig. S3C and S3D). Importantly, CD36-depleted cells present decrease uptake of fluorescently-labeled LDL as revealed by lower DiI-LDL lipid area (Supplementary Fig. S3E). In contrast, a neutralizing antibody against LDL-R had no impact in the LDL-induced migration or lipid droplet accumulation in MDA-MB-231 cells (Supplementary Fig. S3F and S3G). Treatment with SSO abrogated the LDL-induced alterations in mitochondrial mass and network in migrating MDA-MB-231 cells, as determined by relative mtDNA content (Fig. 3F), number of mitochondria expressing TOM20 (Fig. 3G) and HSP60 (Fig. 3H; Supplementary Fig. S3H) and mitochondrial network, quantified as the area occupied by TOM20 mitochondrial particles in the total (phalloidin staining) cell area (Fig. 3I). SSO treatment diminished the proportion of LDL-exposed cells presenting a filamentous mitochondrial network distribution (Fig. 3H; Supplementary Fig. S3I: filamentous network 63.2% in LDL vs. 54.5% in SSO+LDL). Moreover, CD36 knockdown also abolished the LDL-induced increase in mtDNA content in MDA-MB-231 cells (Supplementary Fig. S3J).

Taken together, these results reveal that CD36 is specifically required for the LDL-induced increase in LDL uptake and lipid droplets, migration and mitochondrial network remodeling in migrating MDA-MB-231 cells.

### Lipid Exposure Induces Metabolic and Bioenergetic Dependencies in Breast Cancer Cells

**FIGURE 4:**
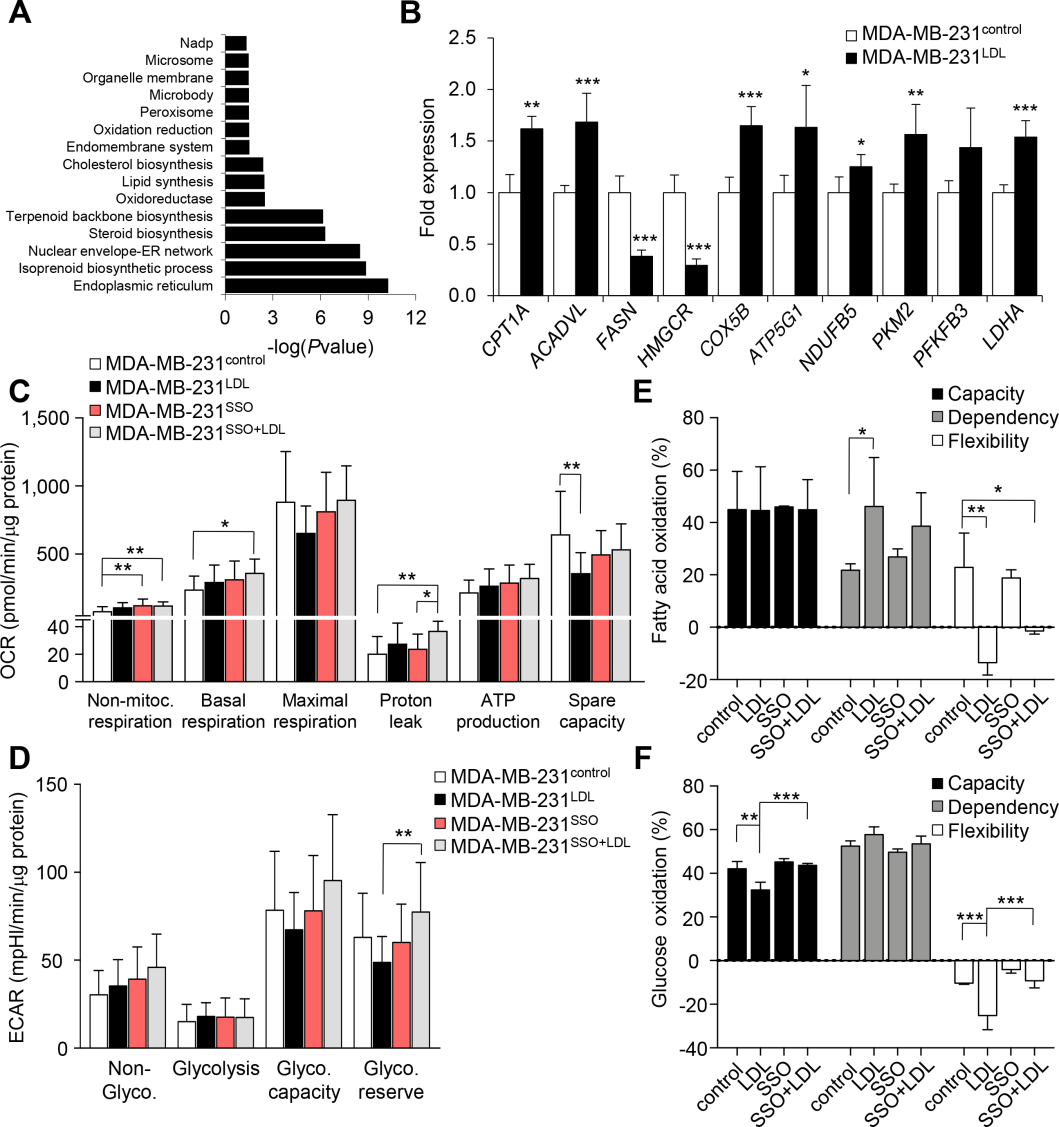
Lipid exposure induces metabolic and bioenergetic dependencies in breast cancer cells. **A,** Gene set enrichment analysis of transcriptomes of MDA-MB-231 control cells compared with LDL-exposed for 48 hours depicting repressed signaling pathways. **B,** qPCR analysis of the relative expression of the indicated genes in untreated (control) or LDL-exposed MDA-MB-231 cells (*n* = 4/5 each). OCR (**C**) and ECAR (**D**) of control or LDL-exposed MDA-MB-231 cells cultured in the absence (vehicle) or presence of SSO for 48 hours (*n* = 12/15 each from three independent experiments). Fuel dependency depicting OCR for FAO (**E**) and glucose oxidation **(F**) of control or LDL-exposed MDA-MB-231 cells cultured in the absence (vehicle) or presence of SSO (*n* = 3 each). Data are presented as mean ± SD. For **A**, statistic cutoff [−*log10 (0.05)*] was applied for Benjamini FDR correction. Data are presented as mean ± SD. *, *P* < 0.05; **, *P* < 0.01; ***, *P* < 0.001.

When FAs are the most abundant energy substrate, mitochondrial bioenergetics and OXPHOS can be tuned by transcriptional regulation of metabolic enzymes and electron transport chain (ETC) complexes (42). To assess whether LDL exposure impacts the bioenergetic program adopted by TNBC cells, we started by analyzing gene expression microarray data from control and 48 hours LDL-exposed MDA-MB-231 cells (19). Gene set enrichment analysis revealed reduced expression of endoplasmic reticulum-related and several metabolic pathways components in LDL-exposed breast cancer cells, with significant decrease in biosynthesis of lipids (steroid, isoprenoid, cholesterol) and membrane structures (nuclear envelope-endoplasmic reticulum (ER) network, endomembrane system, organelle membrane; Fig. 4A). In addition, there was a nonsignificant increase FAO and carbohydrate catabolism (Supplementary Fig. S4A), suggesting that LDL exposure remodels the expression of several metabolic pathways in TNBC cells.

These findings were further validated by qPCR analysis which confirmed increased expression of FAO enzymes (*CPT1A*, *ACADVL)*, decreased expression of lipid biosynthetic enzymes, including the rate-limiting *FASN* and *HMGCR*, and an overall increase expression of bioenergetic enzymes, both OXPHOS (*COX5B*, *ATP5G1*, *NDUFB5*) and glycolytic (*PKM2*, *PFKFB3*, *LDHA*), in LDL-exposed MDA-MB-231 cells (Fig. 4B). To functionally test the reliance on mitochondrial respiration and glycolysis for LDL-induced migration in TNBC cells, we treated MDA-MB-231 cells with oligomycin, an inhibitor of ATP synthase, and 2-DG, a glucose analogue which blocks the glycolytic enzyme hexokinase 2. Treatment with oligomycin produced a dramatic suppression in the migratory capacity of both control and LDL-exposed MDA-MB-231 cells, which was accompanied by an increase in lipid droplets (Supplementary Fig. S4B and S4C). 2-DG prompted a significant, but less pronounced, suppression of the LDL-induced migratory advantage of MDA-MB-231 cells and a reduction in lipid droplets (for both control and LDL-exposed cells; Fig. 4D and E), suggestive of increased usage of stored lipids under glycolytic blockade.

Using the Seahorse extracellular flux analysis, we next performed a series of functional mitochondrial stress tests on cultured MDA-MB-231 cells untreated or LDL exposed in the presence or absence of the CD36 inhibitor SSO. Surprisingly, no significant differences were observed in the OCR between control and LDL-treated MDA-MB-231 cells, including basal and maximal mitochondrial respiration and ATP production (Fig. 4C; Supplementary Fig. S4F). However, compared with untreated cells, LDL-exposed cells present a trend for increased proton leak and a significant decrease in spare capacity (Fig. 4C; *P* = 0.0036), obtained by the difference between the maximal and basal respiration, and which has been associated with increased susceptibility to oxidative stress (43). In addition, LDL treatment did not significantly impact the ECAR of MDA-MB-231 cells, with only a (nonsignificant) decrease in glycolytic reserve (Fig. 4D; Supplementary Fig. S4G). Blockade of CD36 by SSO treatment alone or combined with LDL promoted several OCR bioenergetic adaptations, including increased non-mitochondrial respiration, basal respiration, proton-leak (Fig. 4C; Supplementary Fig. S4F) and glycolytic reserve (Fig. 4D; Supplementary Fig. S4G) suggesting that decreased cellular lipid uptake by CD36 blockade potentiates bioenergetics either by recruitment of intracellular lipids storage (see Fig. 3E) or usage of alternative fuels (e.g., glucose or glutamine). Next, we investigated whether LDL exposure impacts the main energetic fuel preferentially used by TNBC cells by performing substrate dependency assays (Seahorse Mito Fuel Flex Test) based on OCRs response to glutamine-, glucose-, and FAO inhibitors (BPTES, UK5099, and etomoxir, respectively). We observed that LDL-exposed MDA-MB-231 cells present increased FAO dependency and decreased flexibility for mitochondrial respiration (Fig. 4E) and CD36 blockade had no significant impact (Fig. 4E). On the contrary, LDL exposure led to a decrease glucose oxidation capacity and flexibility in MDA-MB-231 cells and CD36 blockade was able to revert this effect (Fig. 4F). Overall, these results suggest that LDL exposure does not impact the main bioenergetic pathway adopted by MDA-MB-231 cells but promotes a dependency on FAs usage for mitochondrial respiration, decreasing the capacity of TNBC cells to adapt to other fuels, such as glucose. Moreover, CD36 is required for the LDL-induced bioenergetic adaptations in the usage of energetic fuels (FAs and glucose) for mitochondrial respiration of MDA-MB-231 cells.

### Lipid-induced Migration of Breast Cancer Cells Rely on FA Transport into the Mitochondria

**FIGURE 5:**
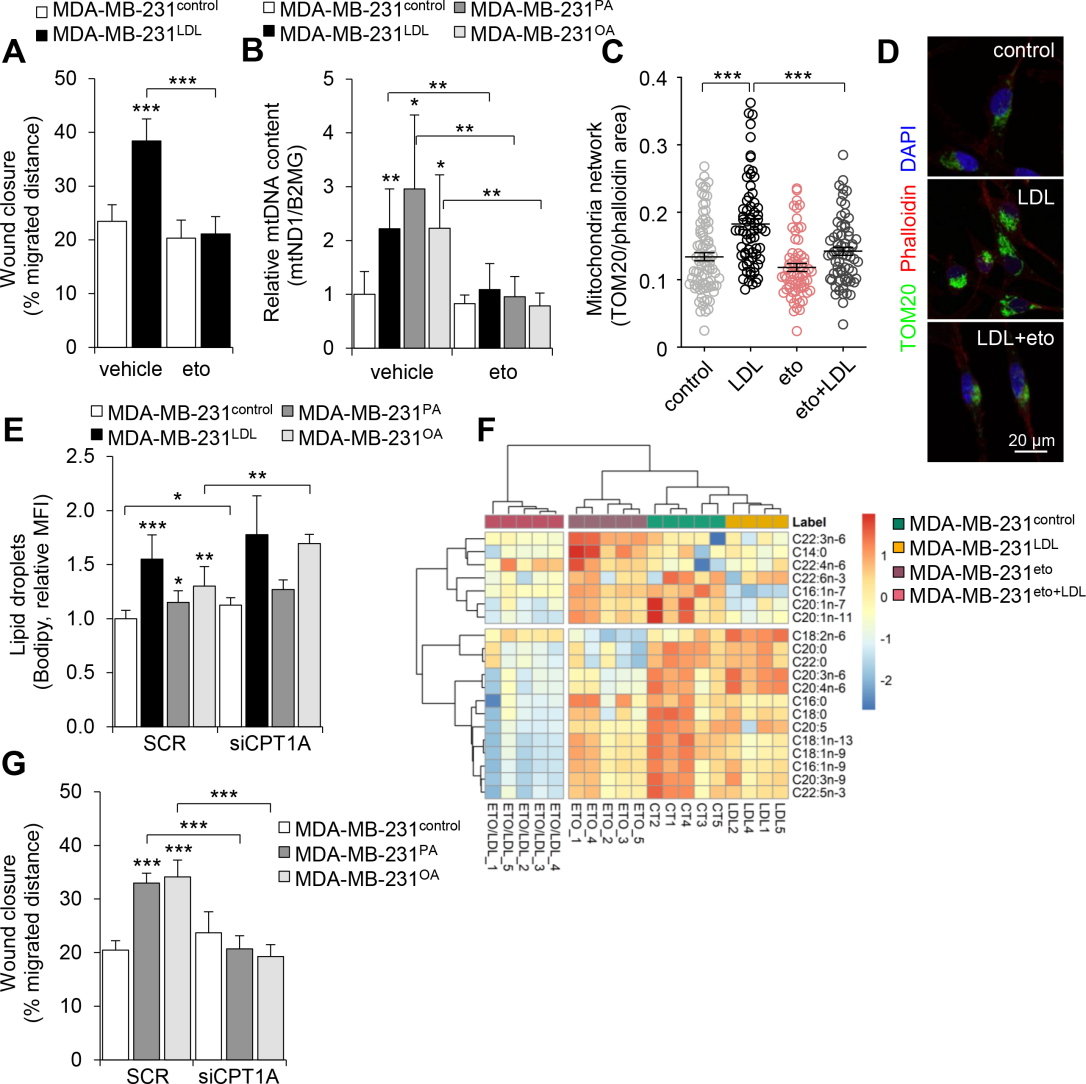
Lipid-induced migration of breast cancer cells rely on FA transport into the mitochondria. **A,** Migratory capacity represented as percentage of wound closure of control or LDL-exposed MDA-MB-231 cells in the absence (vehicle) or presence of etomoxir (eto, 100 μmol/L, *n* = 8 each). **B,** Relative mtDNA content of control, LDL, PA (50 μmol/L), or OA (50 μmol/L)-exposed MDA-MB-231 cells in the absence (vehicle) or presence of etomoxir (eto, 100 μmol/L, *n* = 4/8 each). Mitochondrial network determined as the TOM20 total area over the phalloidin area (**C**) and representative images of immunolabeled (**D**) control or LDL-exposed MDA-MB-231 migrating cells in the absence or presence of etomoxir (eto, 100 μmol/L, *n* = 63/82 cells per condition). TOM20 (green) and phalloidin (red), nuclei are labeled with DAPI (blue). 63× objective, Scale bar, 20 μm (Micrometer). **E,** Quantification of lipid droplets depicted by BODIPY 493/503 (Bodipy) staining depicted as relative median fluorescence intensity (MFI) of siSCR or siCPT1A MDA-MB-231 cells in the absence (control) or presence of LDL, PA (50 μmol/L) or OA (50 μmol/L; *n* = 5/8 each). **F,** Two-dimensional hierarchical clustering heatmap of the FA profiling by GC-MS in mitochondrial extracts isolated from control or LDL-exposed MDA-MB-231 cells in the absence or presence of etomoxir (eto, 100 μmol/L, *n* = 4/5 each). Levels of normalized abundance (log) are shown on the color scale, with number indicating the fold difference from the mean. **G,** Wound closure of siSCR or siCPT1A MDA-MB-231 cells in the absence (control) or presence of PA (75 μmol/L) or OA (75 μmol/L; *n* = 4/8 each). Data are presented as mean ± SD. Each circle in the plot **C** represents individual cell measurement. *, *P* < 0.05; **, *P* < 0.01; ***, *P* < 0.001.

To address the requirement of FAs import into the mitochondria for the LDL-induced aggressive properties of TNBC cells, we used etomoxir to inhibit the carnitine palmitoyltransferase system (CPT1A) that catalyzes the first reaction to shuttle long-chain fatty acids (LCFA) into the mitochondria (11, 44). Etomoxir abrogated the LDL increased migratory capabilities (eto; Fig. 5A), augmented mtDNA content (Fig. 5B) and MitoTracker staining (Supplementary Fig. S5A) in migrating MDA-MB-231 cells, with no major impact in lipid droplet content (Supplementary Fig. S5B). CPT1A blockade by etomoxir produced a dramatic decrease in the number of TOM20–labeled mitochondria but LDL cotreatment was still able to increase the number of mitochondrial particles (Supplementary Fig. S5C). However, cells cotreated with etomoxir and LDL present a significant reduction in the mitochondrial network spread as compared with LDL-treated only (Fig. 5C and D; LDL vs. eto+LDL, *P* < 0.0001). Similarly, siRNA-mediated depletion of CPT1A (Supplementary Fig. S5D) in MDA-MB-231 cells abolished the LDL-induced migration (Supplementary Fig. S5E) and mtDNA content (Supplementary Fig. S5F) and promoted an increase (significant for control cells) in the lipid droplets content (Fig. 5E), probably due to decreased mobilization and usage of FAs in the mitochondria.

These data are consistent with LDL promoting increase FAs trafficking and accumulation into the mitochondria of MDA-MB-231 migrating cells. To directly test this hypothesis, we determined the FA profile by GC-MS of mitochondrial samples isolated from MDA-MB-231 cells exposed to LDL in the absence or presence of etomoxir. Overall, among all the samples we identified 20 different LCFA species (Supplementary Table S2). The most abundant LCFA species in the mitochondrial extracts of MDA-MB-231 cells were PA (C16:0), stearic acid (C18:0), and OA (C18:1n-9), whereas, in the commercial LDL stock (lipoprotein), PA (C16:0) and OA (C18:1n-9) were also among the most represented, together with linoleic acid (C18:2n-6; Supplementary Table S2). Treatment with etomoxir produced significant differences in the mitochondrial FA profile of MDA-MB-231 cells (Fig. 5F). Using two-dimensional hierarchical clustering from univariate analysis, a primary split (in the upper hierarchical dendrogram) shows that the samples clustered independently in two groups: one cluster for the etomoxir and LDL cotreated cells (eto+LDL) and another for the remaining groups (Fig. 5F). The clustering of individual FAs species with respect to their similarity in changes of relative abundances revealed cluster in two groups (a first cluster of seven LCFA and a second group containing 13 LCFA species); both clearly decreased in the eto+LDL group, consistent with our hypothesis. Analysis of individual LCFA normalized abundance revealed significant increase in some species (as linoleic acid, C18:2n-6) in the mitochondria of LDL-exposed MDA-MB-231 cells compared with control (control-green, LDL-yellow; Supplementary Fig. S6) whereas on the contrary, other species (as OA, C18:1n-9 and octadecenoic acid, C18:1n-13) show significant higher abundance in control-treated (Supplementary Fig. S6). Regardless, cotreatment with etomoxir and LDL (eto+LDL-pink; Supplementary Fig. S6) was consistently able to reduce the abundance of those LCFA species as compared with LDL-treated only (Fig. 5F; Supplementary Fig. S6).

To determine the specific contribution of LCFA and their mitochondrial import for the LDL-induced aggressive properties of TNBC cells, we tested the impact of PA and OA that present high relative content in the lipoprotein stock and mitochondrial extracts of MDA-MB-231 cells (Supplementary Table S2) and reduce abundance upon etomoxir and LDL cotreatment compared with LDL only (Fig. 5F; Supplementary Fig. S6). As for LDL, exposure of MDA-MB-231 cells to PA and OA produced an increase in the migratory phenotype (Supplementary Fig. S5G; Fig. 5G), mitochondrial mass, as measured by mtDNA content (Fig. 5B) and lipid droplets (Fig. 5E) that is reversed by CPT1A depletion (Fig. 5G) or etomoxir treatment (Fig. 5B), respectively, with no major impact in lipid droplets for PA and increase storage in OA-treated cells (as observed for the control condition, Fig. 5E). Moreover, as for LDL, PA, and OA-induced migration and internalization by MDA-MB-231 cells requires CD36 (Supplementary Fig. S5H and S5I), as previously shown by others (23). These results suggest that at least part of the LDL impact in TNBC cells migration requires entry of FAs into the mitochondria.

### ROS Downstream of FA Transport into the Mitochondria is Required for the LDL-induced Migration of Breast Cancer Cells

**FIGURE 6:**
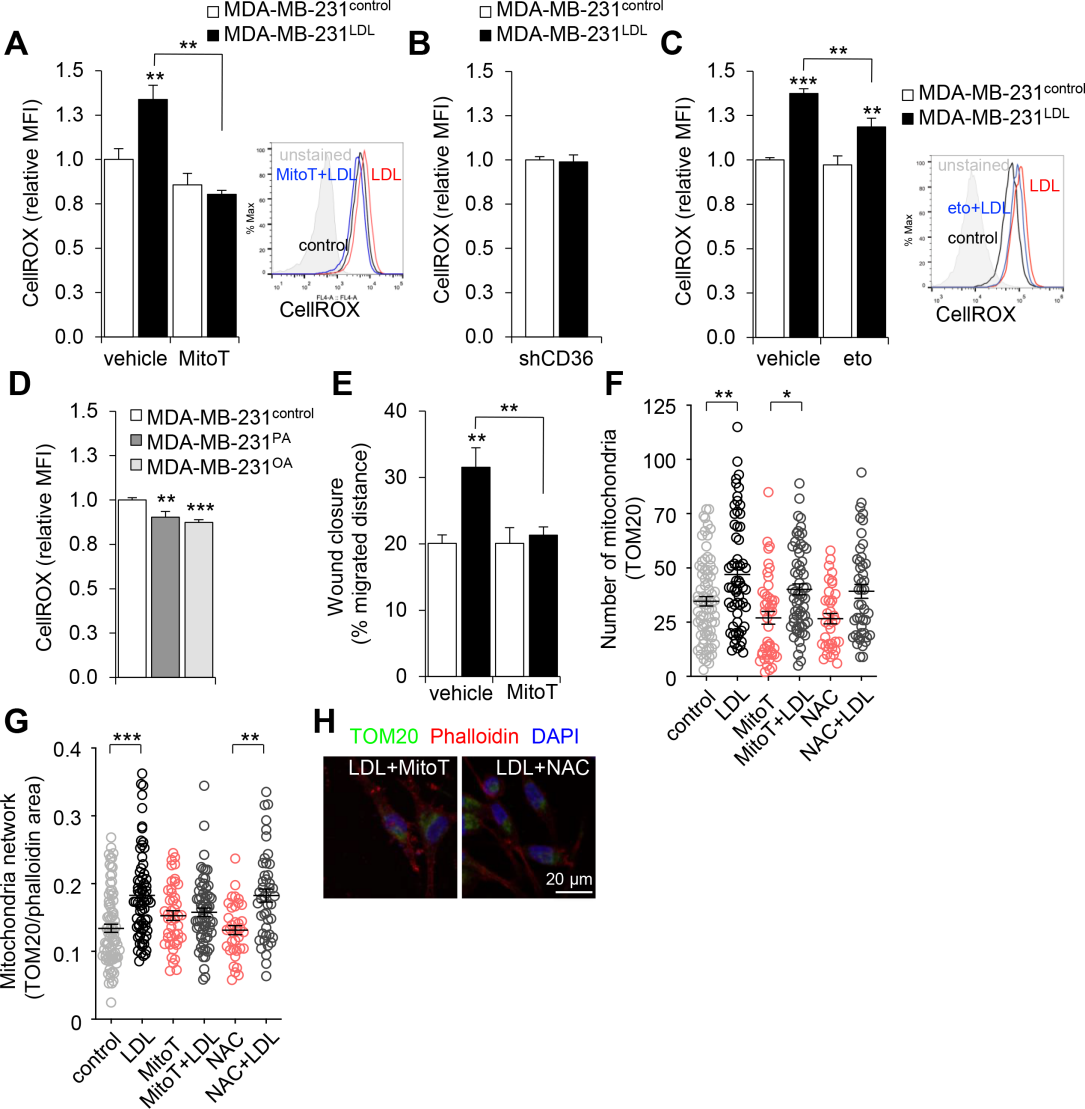
ROS downstream of mitochondria FA entry is required for LDL-induced migration of breast cancer cells. **A,** Flow cytometry quantification of CellROX Deep Red staining depicted as relative median fluorescence intensity (MFI, left) in control or LDL-exposed MDA-MB-231 cells in the absence (vehicle) and presence of MitoTEMPO (MitoT, 100 μmol/L; *n* = 3/6 each) and representative histograms (right). **B,** Flow cytometry quantification of CellROX Deep Red staining depicted as relative MFI in shCD36 control or LDL-exposed MDA-MB-231 cells (*n* = 3/6 each). **C,** Flow cytometry quantification of CellROX Deep Red staining depicted as relative MFI, left) in control or LDL-exposed MDA-MB-231 cells in the absence (vehicle) and presence of etomoxir (eto, 100 μmol/L; *n* = 3/6 each) and representative histograms (right). **D,** Flow cytometry quantification of CellROX Deep Red staining depicted as relative MFI in control, PA (50 μmol/L) or OA (50 μmol/L)-exposed MDA-MB-231 cells (*n* = 4 each). **E,** Migratory capacity represented as percentage of wound closure of control or LDL-exposed MDA-MB-231 cells in the absence (vehicle) or presence of MitoTEMPO (MitoT, 100 μmol/L, *n* = 3 each). Number of TOM20–labeled mitochondria (*n* = 36/78 cells per condition; **F**), mitochondrial network determined as the TOM20 total area over the phalloidin area (*n* = 36/82 cells per condition; **G**), and representative images of immunolabeled (**H**) control or LDL-exposed MDA-MB-231 migrating cells in the absence or presence of NAC (5 mmol/L) or MitoTEMPO (MitoT, 100 μmol/L). TOM20 (green) and phalloidin (red), nuclei are labeled with DAPI (blue). 63x objective, Scale bar, 20 μm (Micrometer). Data are presented as mean ± SD. Each circle in the plot **F** and **G** represents individual cell measurement. *, *P* < 0.05; **, *P* < 0.01; ***, *P* < 0.001.

LDL-exposed MDA-MB-231 cells present several deregulated pathways related to ROS metabolism as repression of oxidoreductase and oxidation reduction (Fig. 4A) and induction of disulfide bond and metabolism of xenobiotics by cytochrome P450 (Supplementary Fig. S4A), and a lower respiratory spare capacity (Fig. 4C), raising the possibility that the mitochondria of LDL-treated MDA-MB-231 cells are exhibit excessive oxidative stress. Indeed, LDL-treated MDA-MB-231 cells present increased levels of cellular ROS, as accessed by CellROX (Fig. 6A, vehicle) in a CD36-dependent manner, as CD36 knockdown (Fig. 6B) or SSO treatment (Supplementary Fig. S7A) blocked the LDL-induced increase in cellular ROS. Moreover, cotreatment with etomoxir led to a significant reduction in the production of ROS by LDL-exposed MDA-MB-231 cells (Fig. 6C; LDL vs. eto+LDL, *P* = 0.0064) which is also observed upon CPT1A depletion (Supplementary Fig. S7B), suggesting that mitochondrial import of LCFA is necessary for the excessive production of ROS. However, treatment with PA and OA alone produce a modest but consistent decrease in ROS formation in MDA-MB-231 cells (Fig. 6D) and no major impact was observed upon CPT1A depletion (Supplementary Fig. S7B), indicating that exposure to individual LCFA species is not sufficient to recapitulate the LDL-induced increase in ROS production.

Consistent with previous studies (45), ROS production induced by LDL was unlikely due to downregulation of antioxidant enzymes (Supplementary Fig. S7C) as LDL-exposed cells present even higher levels of catalase, a peroxisome enzyme that converts hydrogen peroxide to water, probably as an attempt to counterbalance the oxidative stress. LDL-induced increase in ROS was abolished using the mitochondria targeted antioxidant MitoTEMPO (MitoT; Fig. 6A) and the antioxidant NAC (Supplementary Fig. S7D), suggesting that mitochondria contribute, at least in part, for the ROS formation in LDL-exposed MDA-MB-231 cells. Importantly, both mitochondria-specific and cellular antioxidants were able to block the LDL-induced migration (Fig. 6E; Supplementary Fig. S7E) whereas no effect was observed in CD36-depleted MDA-MB-231 cells (Supplementary Fig. S7F). Antioxidant supplementation impacted the mitochondrial network of MDA-MB-231 migrating cells, with NAC abolishing the increase in TOM20–labeled mitochondria (Fig. 6F) and MitoTEMPO blocking the remodeling of mitochondrial network and acquisition of a filamentous distribution (Fig. 6G and H) in LDL-exposed MDA-MB-231 cells.

## Discussion

The role of elevated LDL and dyslipidemia in cancer has been under scrutiny by our lab and others (15–20). We have shown that high LDL in particular promotes breast cancer progression by acting directly on cancer cells inducing proliferation and migration (18, 19) and by suppressing immune responses, namely the action of gamma delta T cells (20). In particular, CD36-mediated metabolic adaptations have been implicated in lipid-dependent breast cancer aggressive phenotypes, including metastasis, resistance to HER2-targeted therapy and modulation of the tumor microenvironment by T-cell dysfunction (21, 23, 46–48). Given the role of lipids in several metabolic processes, most notably in mitochondria (where FAO mainly occurs), here, we exploited the hypothesis that LDL induces breast cancer cells migration by modulating mitochondrial metabolism.

LDL exposure cell-autonomously altered the migratory behavior of TNBC cells and tropism to secondary organs in the zebrafish larvae model, with cells invading organs with peripheral and less vascularized location (Fig. 1; Supplementary Fig. S1; ref. 32). The metastatic cascade is a complex process that involves several sequential steps (49). In the zebrafish larvae, breast cancer cells (of both conditions) were able to undertake the first steps (local invasion into surrounding tissues, intravasation to circulatory system and surviving hematogenous dissemination), as observed in highly vascularized CHT (Fig 1). Invadopodium as cytoplasmic protrusions of transformed cells play a role on extravasating, breaching the endothelial junctions (50), and its formation is cholesterol dependent (51), suggesting that LDL might endow migrating TNBC cells with increase ability to form invadopodia and extravasate to secondary organs.

Our data provide solid evidence for a new mechanistic insight into the impact of LDL in TNBC cells, in particular by establishing CD36, but not LDL-R, as a key requirement for cell migration, uptake of LDL and lipid droplets accumulation, remodeling of mitochondrial network and bioenergetic adaptations in the usage of energetic fuels for respiration, and excessive ROS production. Strikingly, no colocalization between lipid droplets and mitochondria network spread was observed (Fig. 3H). Our data show *in vivo* and *in vitro* that LDL induces cell migration and invasion with concomitant mitochondrial mass alterations (increase number and destabilized cristae) and altered (spread) network distribution throughout the migrating cells, classified here as filamentous; which was extensively validated through a variety of techniques including microscopy (both fixed and live), TEM, flow cytometry, and gene expression analysis. Dietary FA uptake and altered metabolism constitute hallmarks of metastasis (6, 14). In particular, PA and OA have been implicated in the metastatic initiating potential and migratory behavior of CD36-positive breast cancer cells (22, 23). As for LDL, our data revealed that both PA and OA induce TNBC cell migration, increase mitochondrial mass and lipid droplet content in a CD36-dependent manner. This effect was not observed for other mitogenic agents (e.g., LPA), and requires actin cytoskeleton remodeling by the Arp2/3 complex (36). In migrating cancer cells, mitochondria are transported to the leading edges, supporting cytoplasmatic protrusions assembly and function and concomitant migration and invasion (9, 34, 52). In accordance, mitochondria of migrating TNBC cells undergo fission (9, 52), so that it can be transported along the cytoskeleton, and LDL exposure did not significantly alter this parameter.

In macrophages, CD36-dependent import of lipids and accumulation of LCFA in the mitochondria, shift the mitochondrial function from ETC to ROS production and activation of inflammatory patterns (45). In cancer cells, ROS are important secondary messengers that regulate extracellular matrix, cytoskeleton remodeling, and cell motility (8, 53). In our study, transcriptomic and bioenergetic analyses revealed that high lipid levels induced significant alterations in cellular metabolism, rendering TNBC cells dependent on FAs as their main energy source for mitochondrial respiration that results in increased oxidative stress, similar to what is, systemically, seen in hyperlipidemia patients (54). Mechanistically, we show that LDL exposure increases cytoplasmic lipid availability (droplets) and production of ROS in migrating TNBC cells. Our GC-MS lipidomic data are consistent with LDL promoting increased FAs trafficking and accumulation in the mitochondria of TNBC cells, with significant elevation of LCFA species in the mitochondria of LDL-treated (linoleic acid) and reduce abundance of most LCFA species when the CPT1A inhibitor etomoxir was combined (Fig. 5F; Supplementary Fig. S6). LCFA mitochondrial accumulation may contribute to the mitochondrial structural and bioenergetic alterations presented by LDL-exposed TNBC cells, including the cristae defects (Fig. 2F–I), and the higher levels of ROS production (Fig. 6A), as suggested by others (45). In our study, inhibition of CPT1A by etomoxir or genetic depletion (by short-interference) interrupts the LDL-induced LCFA transportation into the mitochondria and excessive ROS formation and was able to block both LDL and individual LCFA (PA, OA)-induced increase in TNBC cell migration and mitochondrial mass, suggesting causality. However, it is not clear how LDL-derived LCFA mitochondrial accumulation leads to ROS production as exposure to individual LCFA (PA or OA) is not sufficient to increase ROS (Fig. 6D), suggesting that simultaneous import of several LCFAs species may be required.

Mitochondrial metabolism and network have been implicated in the acquisition of more aggressive and migratory behavior of cancer cells (9, 10, 12, 55, 56), with mitochondria acting as a signaling hub that goes beyond bioenergetics. Our data reveal that lipid exposure cell-autonomously remodel the usage of mitochondria in migrating TNBC cells from (not only) energy producing to signaling generation, being ROS an important downstream inducer of the cell motility pathways ERK and PI3K/AKT, activated in LDL-exposed migrating TNBC cells (19). Our studies further show that blocking LDL uptake and the resulting lipid entry into the mitochondria via CD36 or CPT1A, respectively, or dumping mitochondrial ROS levels by antioxidants, completely abrogate TNBC cell migration and mitochondria metabolism reprogramming.

Taken together, we reveal novel and detailed mechanistic cellular and metabolic changes underlying the migratory behavior of TNBC cells exposed to systemic lipids, and propose that CD36 and/or ROS signaling may provide a novel therapeutic strategy for subsets of invasive and metastatic breast cancer.

## Supplementary Material

Supplementary Figure S1 LDL-exposed breast cancer cells show differential invasion potential and metastatic tropism to distant sites in xenotransplanted zebrafish larvae at 4dpi (6dpf). Related to Fig. 1.

Supplementary Figure S2 LDL exposure induces mitochondrial network spread distribution and destabilized cristae in migrating breast cancer cells. Related to Fig. 2

Supplementary Figure S3 LDL-induced breast cancer cell migration is mediated by the fatty acid transporter CD36. Related to Fig. 3

Supplementary Figure S4 Lipid exposure induces metabolic and bioenergetic dependencies in breast cancer cells. Related to Fig. 4

Supplementary Figure S5 LDL-induced migratory behavior of TNBC cells relies on fatty acid transport into the mitochondria. Related to Fig. 5

Supplementary Figure S6 LDL-induced migratory behavior of TNBC cells relies in fatty acid transport into the mitochondria. Related to Fig. 5

Supplementary Figure S7 ¬Reactive oxygen species formation downstream of mitochondria fatty acid entry is required for LDL-induced migration of breast cancer cells. Related to Fig. 6

Supplementary Figure S8 Uncropped membranes relative to the western blot for Drp1, Mfn1, Mfn2 and b-ACTIN proteins displayed in Fig. 2J

Supplementary Table S1 List of primers used for measurements of gene expression levels and mtDNA content by quantitative real-time PCR.

Supplementary Table S2 Fatty acid profile in relative content (%). Qualitative analysis was calculated by dividing each raw area by the sum of total raw areas. Dash (‘-‘) represents lipid species that were not identified in the sample. Legend: CTR (control), ETO (etomoxir, 100 µM).
