# Supplementary material for: Mitochondrial Metabolism Drives Low-density Lipoprotein-induced Breast Cancer Cell Migration
Source: Cancer Res Commun. 2023 Apr 26;3(4):709–24. doi: 10.1158/2767-9764.CRC-22-0394 (PMC10132314; doi:10.1158/2767-9764.CRC-22-0394)
Supplement: Supplementary Figure S1 — LDL-exposed breast cancer cells show differential invasion potential and metastatic tropism to distant sites in xenotransplanted zebrafish larvae at 4dpi (6dpf). Related to Fig. 1. [file crc-22-0394-s01.pdf]

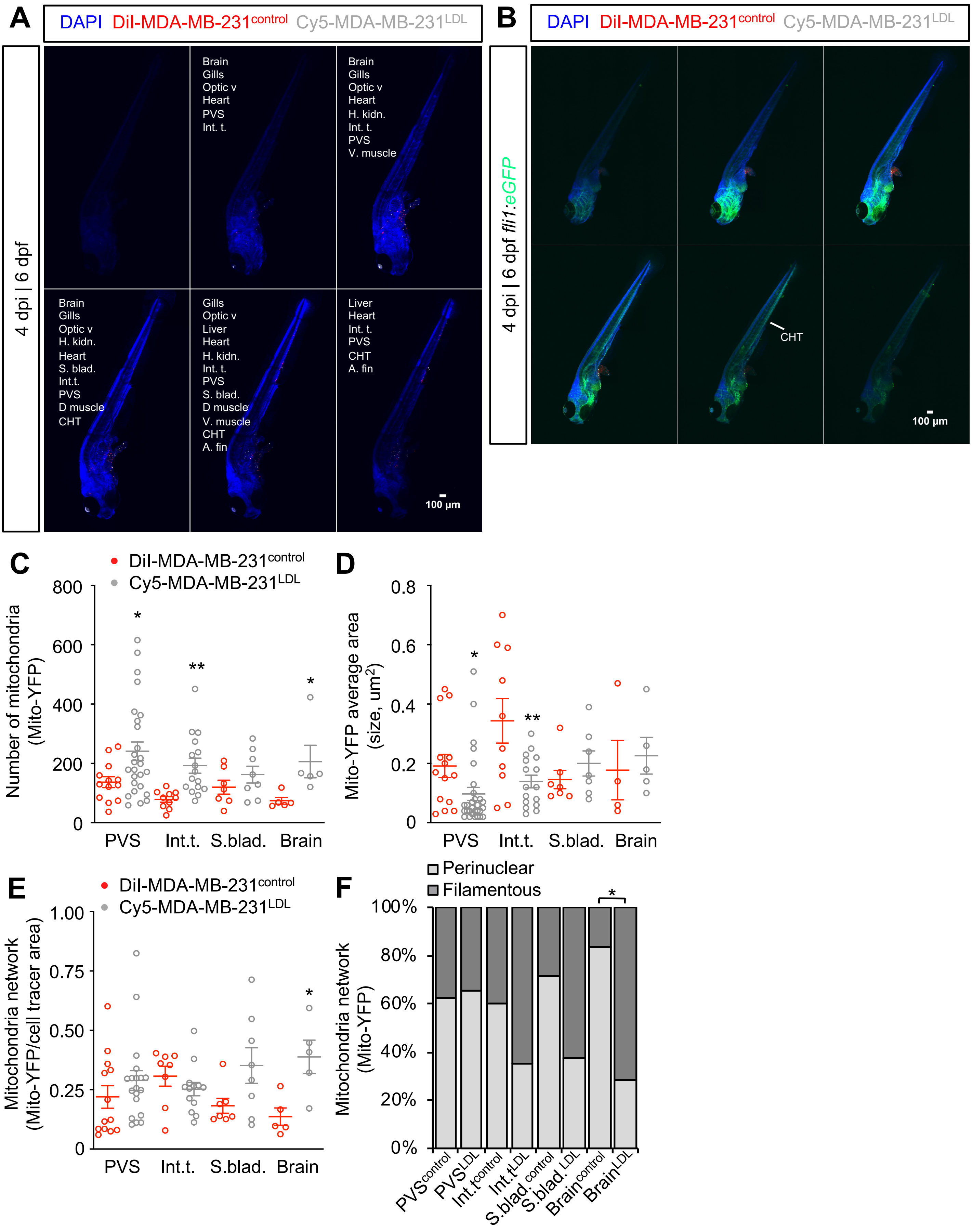
A
DAPI DiI-MDA-MB-231$^{control}$ Cy5-MDA-MB-231$^{LDL}$
4 dpi | 6 dpf
Brain
Gills
Optic v
Heart
PVS
Int. t.
Brain
Gills
Optic v
Heart
H. kidn.
Int. t.
PVS
V. muscle
Brain
Gills
Optic v
H. kidn.
Heart
S. blad.
Int.t.
PVS
D muscle
CHT
Gills
Optic v
Liver
Heart
H. kidn.
Int. t.
PVS
S. blad.
D muscle
V. muscle
CHT
A. fin
Liver
Heart
Int. t.
PVS
CHT
A. fin
100 μm
B
DAPI DiI-MDA-MB-231$^{control}$ Cy5-MDA-MB-231$^{LDL}$
4 dpi | 6 dpf fli1:eGFP
CHT
100 μm
C
DiI-MDA-MB-231$^{control}$
Cy5-MDA-MB-231$^{LDL}$
Number of mitochondria (Mito-YFP)
800
600
400
200
0
*
**
*
PVS Int.t. S.blad. Brain
D
Mito-YFP average area (size, um$^2$)
0.8
0.6
0.4
0.2
0
*
**
PVS Int.t. S.blad. Brain
E
DiI-MDA-MB-231$^{control}$
Cy5-MDA-MB-231$^{LDL}$
Mitochondria network (Mito-YFP/cell tracer area)
1.00
0.75
0.50
0.25
0
*
PVS Int.t. S.blad. Brain
F
Perinuclear
Filamentous
Mitochondria network (Mito-YFP)
100%
80%
60%
40%
20%
0%
*
PVS$^{control}$
PVS$^{LDL}$
Int.t$^{control}$
Int.t$^{LDL}$
S.blad.$^{control}$
S.blad.$^{LDL}$
Brain$^{control}$
Brain$^{LDL}$

**Supplementary Figure S1. LDL-exposed breast cancer cells show differential invasion potential and metastatic tropism to distant sites in xenotransplanted zebrafish larvae at 4dpi (6dpf). (A)** Representative images of whole zebrafish tile in xenotransplanted larvae at 2dpf and analyzed at 4dpi (6dpf) with invaded organs, as detailed in each image, by DiI-labelled MDA-MB-231 control (red) and Cy5-labelled LDL-exposed (grey) cells. (**B)** Representative images of whole DiI-labelled MDA-MB-231 control (red) and Cy5-labelled LDL-exposed (grey) xenotransplanted Tg (fli1:eGFP) zebrafish tile (4dpi and 6dpf) with endothelial fli1:eGFP expression (green). Images were captured with a spinning disk inverted confocal microscope Zeiss Cell Observer SD. Nuclei staining with DAPI is in blue. Scale bar, 100 µm. (**C-E)** Total number (**C**), average area (**D**) and mitochondria network (**E**) determined as the Mito-YFP area by the cell tracer DiI or Cy5 area, for control and LDL cells respectively, in control and LDL-exposed MDA-MB-231 cells quantified in the indicated organs of xenotransplanted imunolabelled zebrafish larvae (PVS, n=13/26; Int. t., n=10/16; S. blad., n=7/8, Brain, n=5 cells). (**F)** Chart representing the Mito-YFP-labelled mitochondrial network distribution of control and LDL-exposed MDA-MB-231 cells in the indicated organs (PVS, n=13/26; Int. t., n=10/16; S. blad., n=7/8, Brain, n=5 cells) from xenotransplated zebrafish larvae.

Legend: A. (anal), CHT (caudal hematopoietic tissue), D. (dorsal), H. kidn. (head kidney), Int. t. (intestinal tract), Optic v. (optic vesicle), PVS (perivitelline space), S. blad. (swim bladder), V. (ventral). Data are presented as mean ± s.d. Each circle in the plot (C-E) represents individual cell measurement. * $p<0.05$, ** $p<0.01$.
