# Supplementary material for: Mitochondrial Metabolism Drives Low-density Lipoprotein-induced Breast Cancer Cell Migration
Source: Cancer Res Commun. 2023 Apr 26;3(4):709–24. doi: 10.1158/2767-9764.CRC-22-0394 (PMC10132314; doi:10.1158/2767-9764.CRC-22-0394)
Supplement: Supplementary Figure S2 — LDL exposure induces mitochondrial network spread distribution and destabilized cristae in migrating breast cancer cells. Related to Fig. 2 [file crc-22-0394-s02.pdf]

Nóbrega-Pereira S_Fig. S2

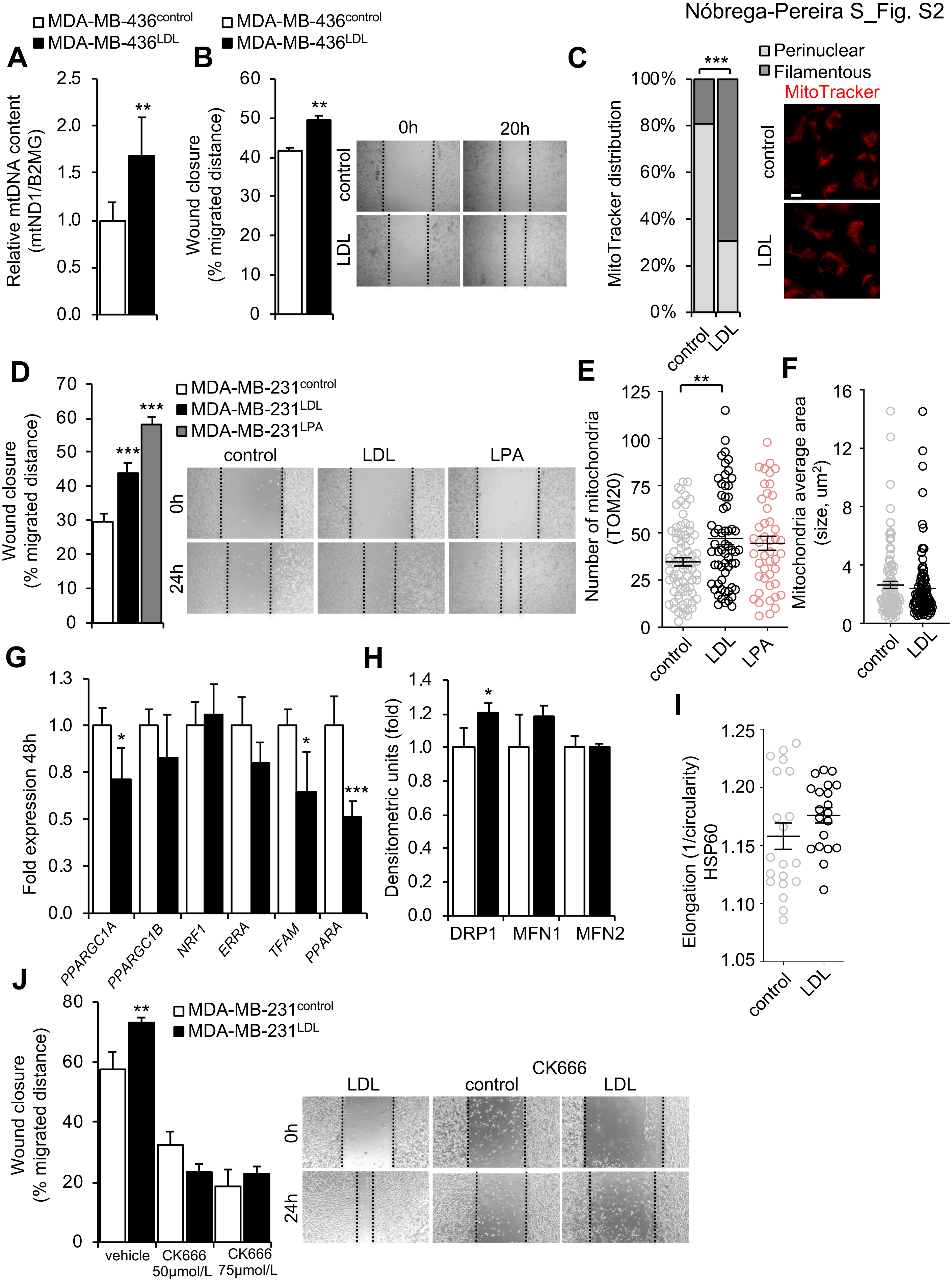

Supplementary Figure S2. LDL exposure induces mitochondrial network spread distribution and destabilized cristae in migrating breast cancer cells. (**A)** Mitochondrial DNA (mtDNA) content accessed by qPCR analysis of the human mitochondrial ND1 gene relative to the nuclear β2-microglobulin gene in DNA samples from untreated (control) or LDL-exposed MDA-MB-436 cells (n=5 each). (**B)** Wound closure of control or LDL-exposed MDA-MB-436 cells (n=3 each) and representative images of wounds at 0h and 20h by optical microscopy (4x objective). (**C)** Chart representing MitoTracker Deep Red live staining of mitochondrial network distribution of control and LDL-exposed migrating MDA-MB-231 cells (n=42 and n=39 cells, respectively) and representative images of mitochondrial network distribution acquired in an inverted fluorescent Zeiss Cell Observer Microscope (63x objective, scale bar 20 µm). (**D)** Wound closure of control, LDL or LPA-exposed MDA-MB-231 cells (n=4 each) and representative images of wounds at 0h and 20h by optical microscopy (4x objective). **(E)** Number of TOM20 labelled mitochondria in control, LDL or LPA-exposed MDA-MB-231 migrating cells (n=45/78 cells per condition). (**F)** Mitochondria average area from TEM imaged sections in control and LDL-exposed migrating MDA-MB-231 cells (n=99 cells). (**G)** qPCR analysis of the relative expression of the indicated genes in untreated (control) or LDL-exposed MDA-MB-231 cells (n=4/5 each). (**H)** Quantification of western blot densitometric units for DRP1, MFN1 and MFN2 protein expression corrected to β-ACTIN (related to Fig. 2J). **(I)** Mean elongation of HSP60-labelled mitochondria number in control and LDL-exposed migrating MDA-MB-231 cells (n=20 each) in the wound-healing assay. (**J)** Wound closure of control or LDL-exposed MDA-MB-231 cells in the absence (vehicle) or presence of CK666 (50 or 75 µmol/L, n=4 each) and representative images of wound closure at 0h and 24h by optical microscopy (4x objective).
Data are presented as mean ± s.d. Each circle in the plot (E, F, I) represents individual cell measurement. * $p<0.05$, ** $p<0.01$, *** $p<0.001$.
