# Supplementary material for: Mitochondrial Metabolism Drives Low-density Lipoprotein-induced Breast Cancer Cell Migration
Source: Cancer Res Commun. 2023 Apr 26;3(4):709–24. doi: 10.1158/2767-9764.CRC-22-0394 (PMC10132314; doi:10.1158/2767-9764.CRC-22-0394)
Supplement: Supplementary Figure S3 — LDL-induced breast cancer cell migration is mediated by the fatty acid transporter CD36. Related to Fig. 3 [file crc-22-0394-s03.pdf]

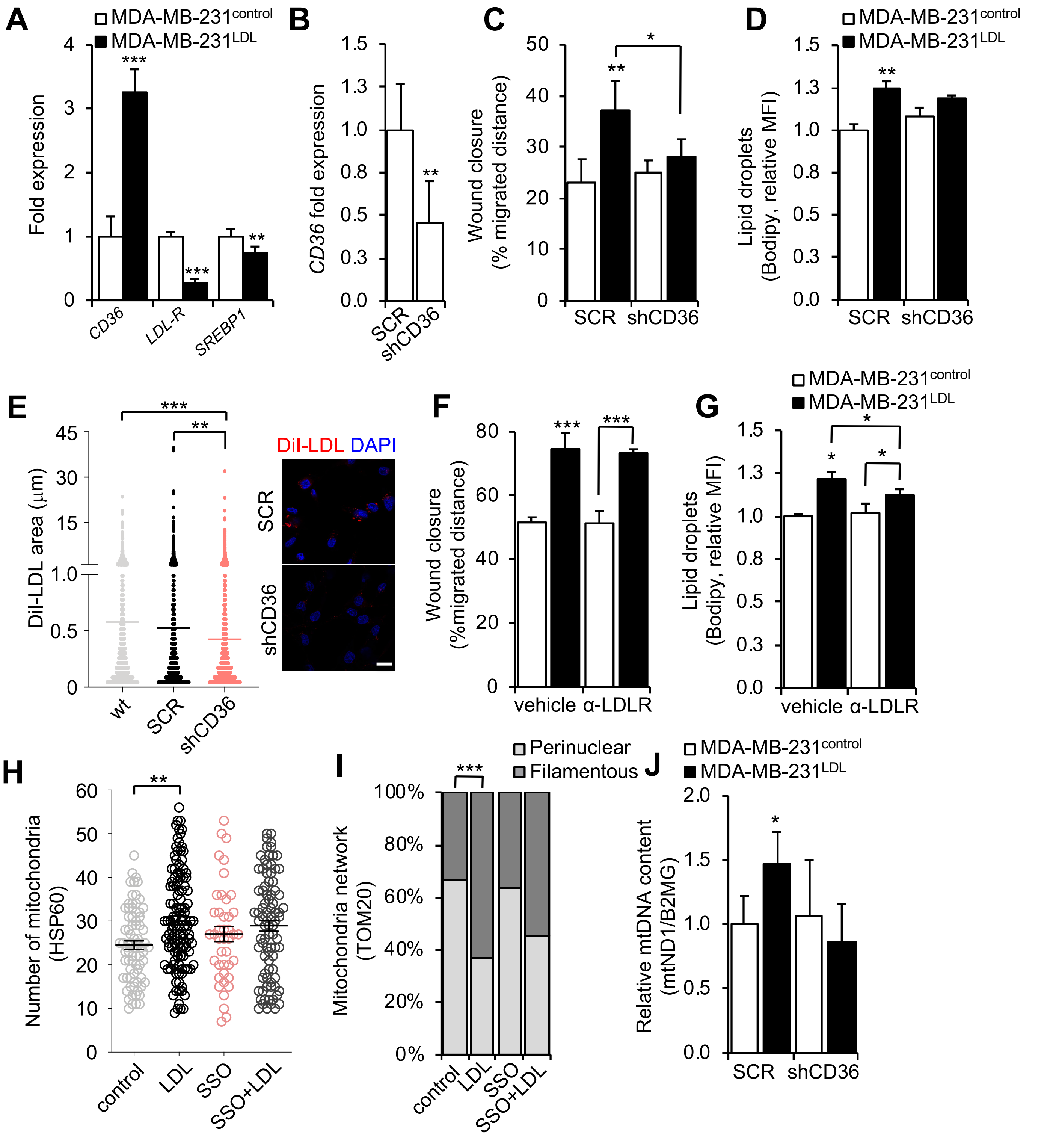
A
MDA-MB-231control
MDA-MB-231LDL
Fold expression
CD36
LDL-R
SREBP1
B
CD36 fold expression
SCR
shCD36
C
Wound closure (% migrated distance)
SCR shCD36
D
Lipid droplets (Bodipy, relative MFI)
E
DiI-LDL area (µm)
wt
SCR
shCD36
DiI-LDL DAPI
F
Wound closure (%migrated distance)
vehicle α-LDLR
G
H
Number of mitochondria (HSP60)
control
LDL
SSO
SSO+LDL
I
Perinuclear
Filamentous
Mitochondria network (TOM20)
J
Relative mtDNA content (mtND1/B2MG)

Supplementary Figure S3. LDL-induced breast cancer cell migration is mediated by the fatty acid transporter CD36. (A) qPCR analysis of the relative expression of the indicated genes in untreated (control) or LDL-exposed MDA-MB-231 cells (n=4/5 each). **(B)** qPCR analysis of the *CD36* relative expression in untreated shSCR and shCD36 MDA-MB-231 cells (n=5/6 each)**. (C)** Wound closure of shSCR or shCD36 MDA-MB-231 cells in the absence (control) or presence of LDL (n=5/7 each). (**D)** Flow cytometry quantification of BODIPY 493/503 (Bodipy) staining depicted as relative median fluorescence intensity (MFI) of untreated (control) or LDL-exposed shSCR and shCD36 MDA-MB-231 cells (n=4). **(E)** DiI-LDL lipid area in LDL-exposed wild-type (wt) (n=2402 lipid particles from 282 cells), shSCR (n=2722 lipid particles from 359 cells) or shCD36 (n=5925 lipid particles from 477 cells) MDA-MB-231 cells after 24h acquired in an inverted fluorescent Zeiss Cell Scanning Confocal Microscope and representative images (40x objective, scale bar 20 µm).(**F-G)** Wound closure (**F**) and flow cytometry quantification of lipid droplets by BODIPY 493/503 (Bodipy) staining (**G**) of untreated (control) or LDL-exposed MDA-MB-231 cells alone (vehicle) or in the presence of an anti-human LDLR antibody (n=4). (**H)** Number of HSP60 labelled mitochondria in control or LDL-exposed migrating MDA-MB-231 cells in the absence (vehicle) or presence of SSO (n=42/119 cells per condition). (**I)** Chart representing TOM20-labelled mitochondrial network distribution of control or LDL-exposed MDA-MB-231 migrating cells in the absence or presence of SSO (n=44/87 cells per condition). (**J)** Relative mitochondrial DNA (mtDNA) content from control (untreated) or LDL-exposed shSCR and shCD36 MDA-MB-231 cells (n=4/7 each).

Data are presented as mean ± s.d. Each circle in the plot (E, H) represents individual lipid particles (E) or cell measurement (H). * $p<0.05$, ** $p<0.01$, *** $p<0.001$.
