# Supplementary material for: Mitochondrial Metabolism Drives Low-density Lipoprotein-induced Breast Cancer Cell Migration
Source: Cancer Res Commun. 2023 Apr 26;3(4):709–24. doi: 10.1158/2767-9764.CRC-22-0394 (PMC10132314; doi:10.1158/2767-9764.CRC-22-0394)
Supplement: Supplementary Figure S4 — Lipid exposure induces metabolic and bioenergetic dependencies in breast cancer cells. Related to Fig. 4 [file crc-22-0394-s04.pdf]

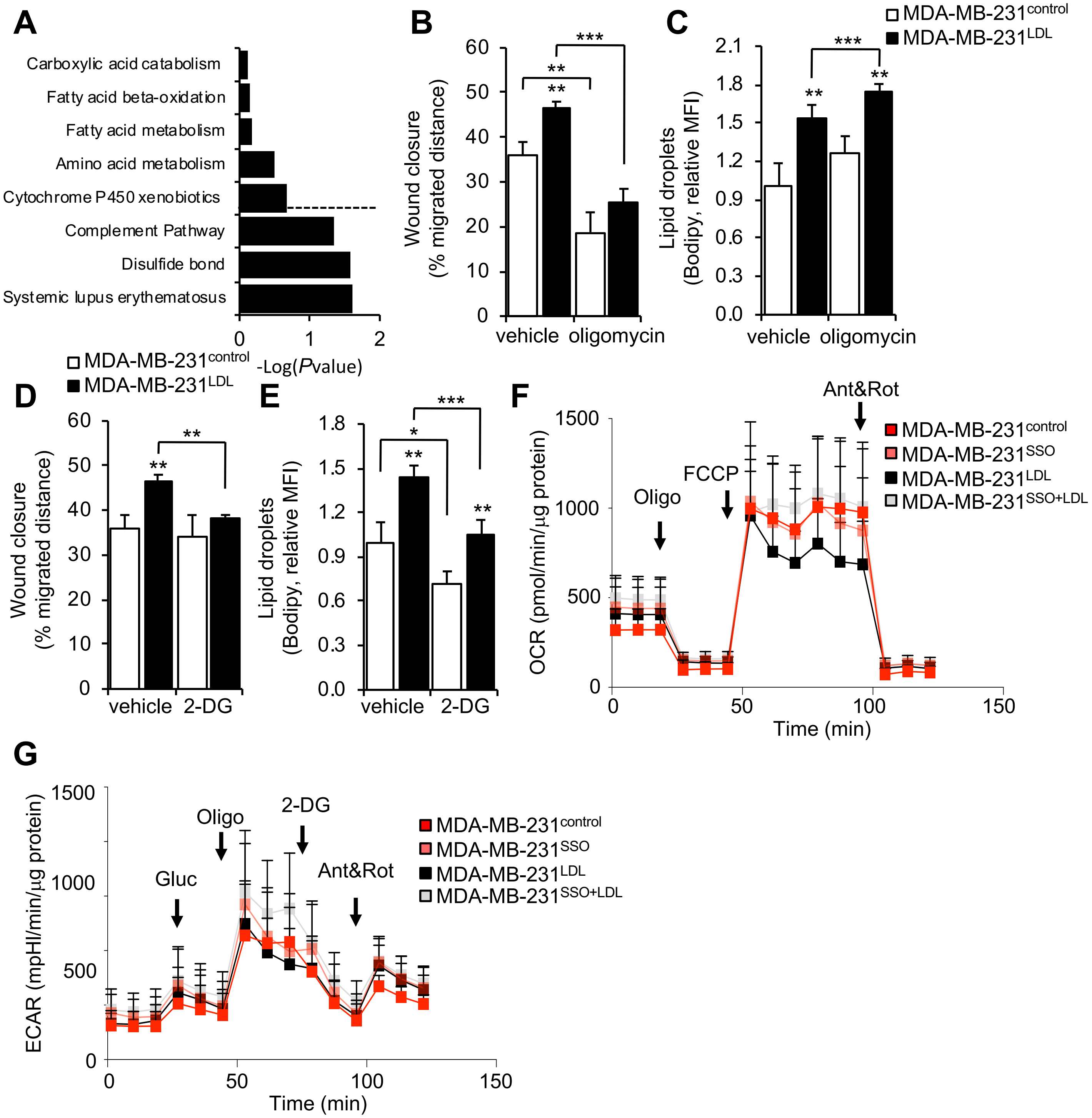
A
Carboxylic acid catabolism
Fatty acid beta-oxidation
Fatty acid metabolism
Amino acid metabolism
Cytochrome P450 xenobiotics
Complement Pathway
Disulfide bond
Systemic lupus erythematosus
0 1 2
-Log(Pvalue)
B
Wound closure (% migrated distance)
vehicle oligomycin
C
Lipid droplets (Bodipy, relative MFI)
vehicle oligomycin
MDA-MB-231control
MDA-MB-231LDL
D
Wound closure (% migrated distance)
vehicle 2-DG
E
Lipid droplets (Bodipy, relative MFI)
vehicle 2-DG
F
OCR (pmol/min/µg protein)
Oligo
FCCP
Ant&Rot
Time (min)
MDA-MB-231control
MDA-MB-231SSO
MDA-MB-231LDL
MDA-MB-231SSO+LDL
G
ECAR (mpHl/min/µg protein)
Gluc
Oligo
2-DG
Ant&Rot
Time (min)
MDA-MB-231control
MDA-MB-231SSO
MDA-MB-231LDL
MDA-MB-231SSO+LDL

Supplementary Figure S4. Lipid exposure induces metabolic and bioenergetic dependencies in breast cancer cells. **(A)** Gene set enrichment analysis (GSEA) of transcriptomes of MDA-MB-231 control cells compared to LDL-exposed for 48h depicting induced signaling pathways. (**B-E)** Wound closure (**B, D**) and flow cytometry quantification of lipid droplets depicted by BODIPY 493/503 (Bodipy) staining as relative median fluorescence intensity (MFI) (**C, E**) of control or LDL-exposed MDA-MB-231 cells in the absence (vehicle) or presence of oligomycin (2 µmol/L, n=4 each) (**B, C**) or 2-DG (2 mmol/L, n=4 each) (**D, E**). **(F-G)** Oxygen consumption rate (OCR) (**F**) and Extracellular acidification rate (ECAR) (**G**) of control or LDL-exposed MDA-MB-231 cells cultured in the absence (vehicle) or presence of SSO for 48h (n=12/15 each from 3 independent experiments).
Data are presented as mean ± s.d. Statistical analysis was performed by One-way ANOVA with multiple comparison. For (A), statistic cut-off [-*log10 (0.05)*] was applied for Benjamini false discovery rate (FDR) correction. Data are presented as mean ± s.d. * $p<0.05$, ** $p<0.01$, *** $p<0.001$.
