# Supplementary material for: Mitochondrial Metabolism Drives Low-density Lipoprotein-induced Breast Cancer Cell Migration
Source: Cancer Res Commun. 2023 Apr 26;3(4):709–24. doi: 10.1158/2767-9764.CRC-22-0394 (PMC10132314; doi:10.1158/2767-9764.CRC-22-0394)
Supplement: Supplementary Figure S5 — LDL-induced migratory behavior of TNBC cells relies on fatty acid transport into the mitochondria. Related to Fig. 5 [file crc-22-0394-s05.pdf]

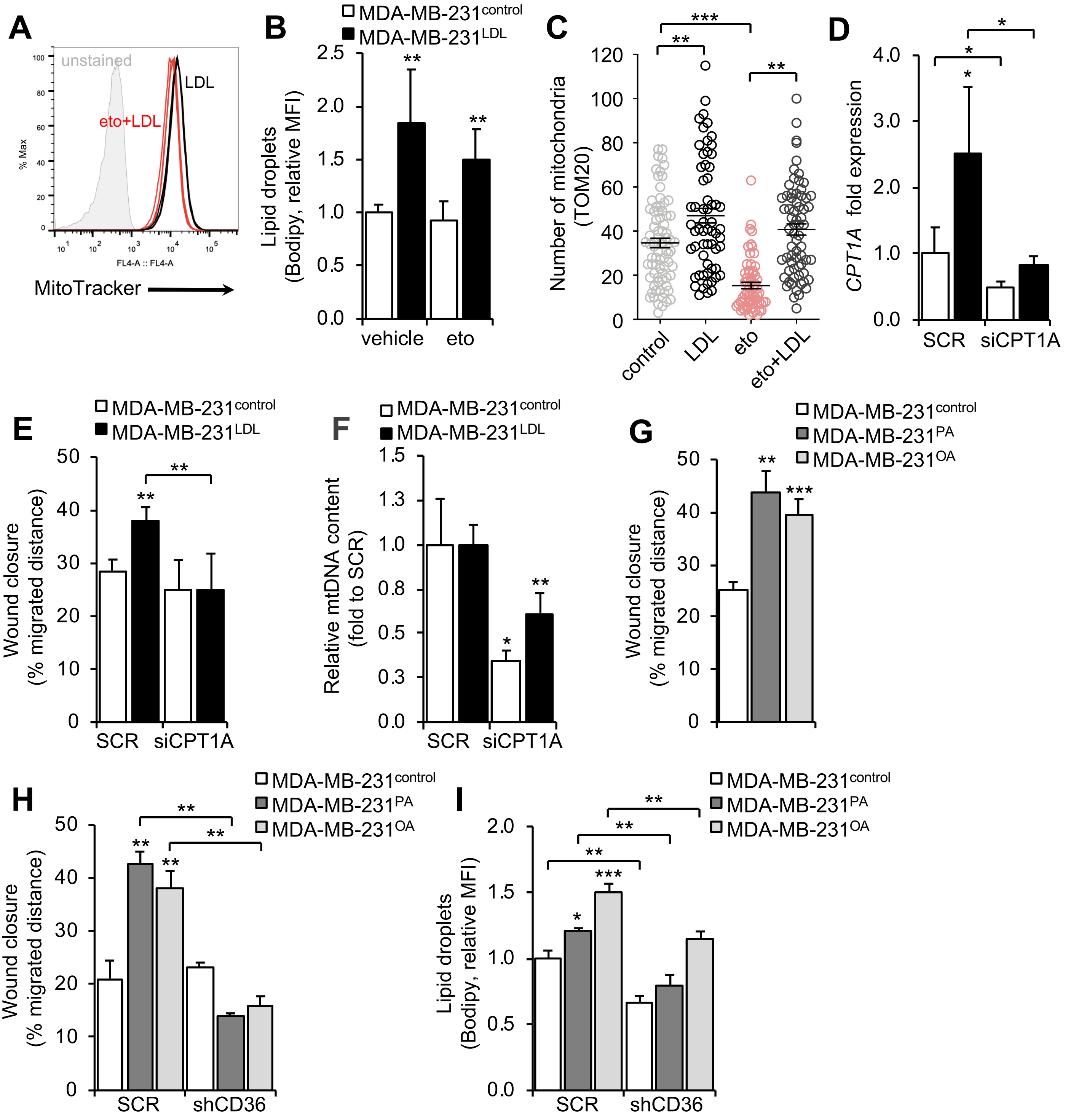
A
unstained
LDL
eto+LDL
% Max
FL4-A :: FL4-A
MitoTracker
B
MDA-MB-231control
MDA-MB-231LDL
Lipid droplets (Bodipy, relative MFI)
vehicle
eto
C
Number of mitochondria (TOM20)
control
LDL
eto
eto+LDL
D
CPT1A fold expression
SCR
siCPT1A
E
MDA-MB-231control
MDA-MB-231LDL
Wound closure (% migrated distance)
SCR
siCPT1A
F
MDA-MB-231control
MDA-MB-231LDL
Relative mtDNA content (fold to SCR)
SCR
siCPT1A
G
MDA-MB-231control
MDA-MB-231PA
MDA-MB-231OA
Wound closure (% migrated distance)
H
MDA-MB-231control
MDA-MB-231PA
MDA-MB-231OA
Wound closure (% migrated distance)
SCR
shCD36
I
MDA-MB-231control
MDA-MB-231PA
MDA-MB-231OA
Lipid droplets (Bodipy, relative MFI)
SCR
shCD36

Supplementary Figure S5. LDL-induced migratory behavior of TNBC cells relies on fatty acid transport into the mitochondria. (**A)** Flow cytometry MitoTracker Deep Red staining (representative histograms) of control or LDL-exposed MDA-MB-231 cells in the absence (vehicle) or presence of etomoxir (eto, 100 μmol/L). (**B)** Quantification of lipid droplets depicted by BODIPY 493/503 (Bodipy) staining depicted as relative median fluorescence intensity (MFI) in control or LDL-exposed MDA-MB-231 cells in the absence (vehicle) or presence of etomoxir (eto, 100 μmol/L, n=6/7 each). **(C)** Number of TOM20 labelled mitochondria in control or LDL-exposed migrating MDA-MB-231 cells in the absence or presence of etomoxir (eto, 100 μmol/L, n=61/78 cells per condition). (**D)** qPCR analysis of the *CPT1A* relative expression in control or LDL-exposed siSCR and siCPT1A MDA-MB-231 cells (n=3/5 each). **(E)** Wound closure of siSCR or siCPT1A MDA-MB-231 cells in the absence (control) or presence of LDL (n=3/7 each). **(F)** Relative mitochondrial DNA (mtDNA) content represented as fold to SCR of MDA-MB-231 cells in the absence (control) or presence of LDL (n=3/4 each). (**G)** Wound closure of control, palmitic acid (PA, 50 μmol/L) or oleic acid (OA, 50 μmol/L)-exposed MDA-MB-231 cells (n=4/5 each). (**H)** Wound closure of shSCR or shCD36 MDA-MB-231 cells in the absence (control) or presence of palmitic acid (PA, 75 μmol/L) or oleic acid (OA, 75 μmol/L) (n=3 each). (**I)** Quantification of lipid droplets depicted by BODIPY 493/503 (Bodipy) staining depicted as relative median fluorescence intensity (MFI) in the absence (control) or presence of palmitic acid (PA, 75 μmol/L) or oleic acid (OA, 75 μmol/L) (n=3/4 each). Data are presented as mean ± s.d. Each circle in the plot (C) represents individual cell measurement. * p<0.05, ** p<0.01, *** p<0.001.
