# Supplementary material for: Mitochondrial Metabolism Drives Low-density Lipoprotein-induced Breast Cancer Cell Migration
Source: Cancer Res Commun. 2023 Apr 26;3(4):709–24. doi: 10.1158/2767-9764.CRC-22-0394 (PMC10132314; doi:10.1158/2767-9764.CRC-22-0394)
Supplement: Supplementary Figure S6 — LDL-induced migratory behavior of TNBC cells relies in fatty acid transport into the mitochondria. Related to Fig. 5 [file crc-22-0394-s06.pdf]

Nóbrega-Pereira S_Fig.S6

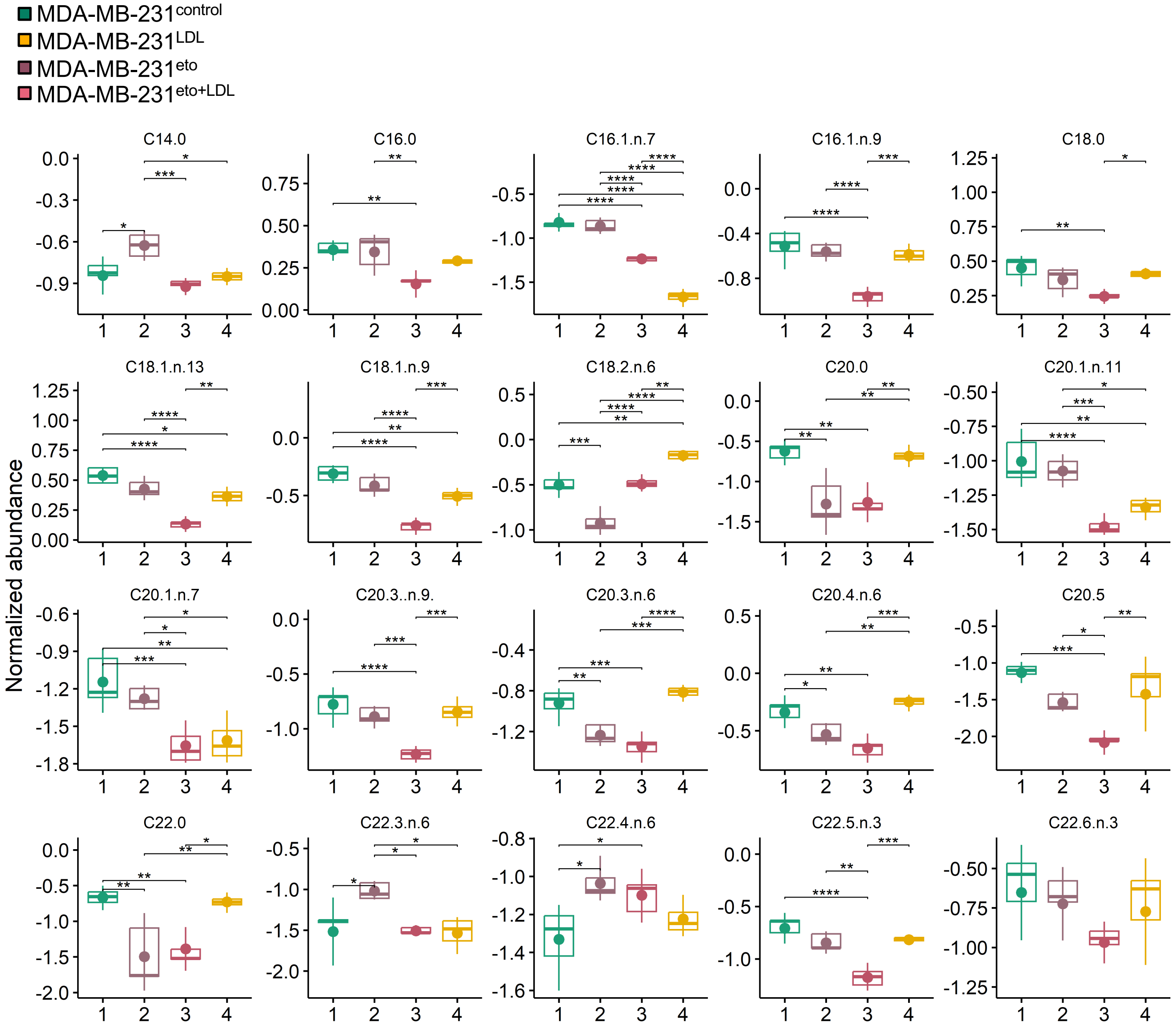

Supplementary Figure S6. LDL-induced migratory behavior of TNBC cells relies in fatty acid transport into the mitochondria. Normalized abundance (log) for each fatty acid by GC-MS identified in mitochondrial extracts isolated from control or LDL-exposed MDA-MB-231 cells in the absence or presence of etomoxir (eto, 100 µmol/L, n=4/5 each).

Data are presented as mean ± s.d. * $p<0.05$, ** $p<0.01$, *** $p<0.001$, **** $p<0.0001$.
