# Supplementary material for: Mitochondrial Metabolism Drives Low-density Lipoprotein-induced Breast Cancer Cell Migration
Source: Cancer Res Commun. 2023 Apr 26;3(4):709–24. doi: 10.1158/2767-9764.CRC-22-0394 (PMC10132314; doi:10.1158/2767-9764.CRC-22-0394)
Supplement: Supplementary Figure S7 — ¬Reactive oxygen species formation downstream of mitochondria fatty acid entry is required for LDL-induced migration of breast cancer cells. Related to Fig. 6 [file crc-22-0394-s07.pdf]

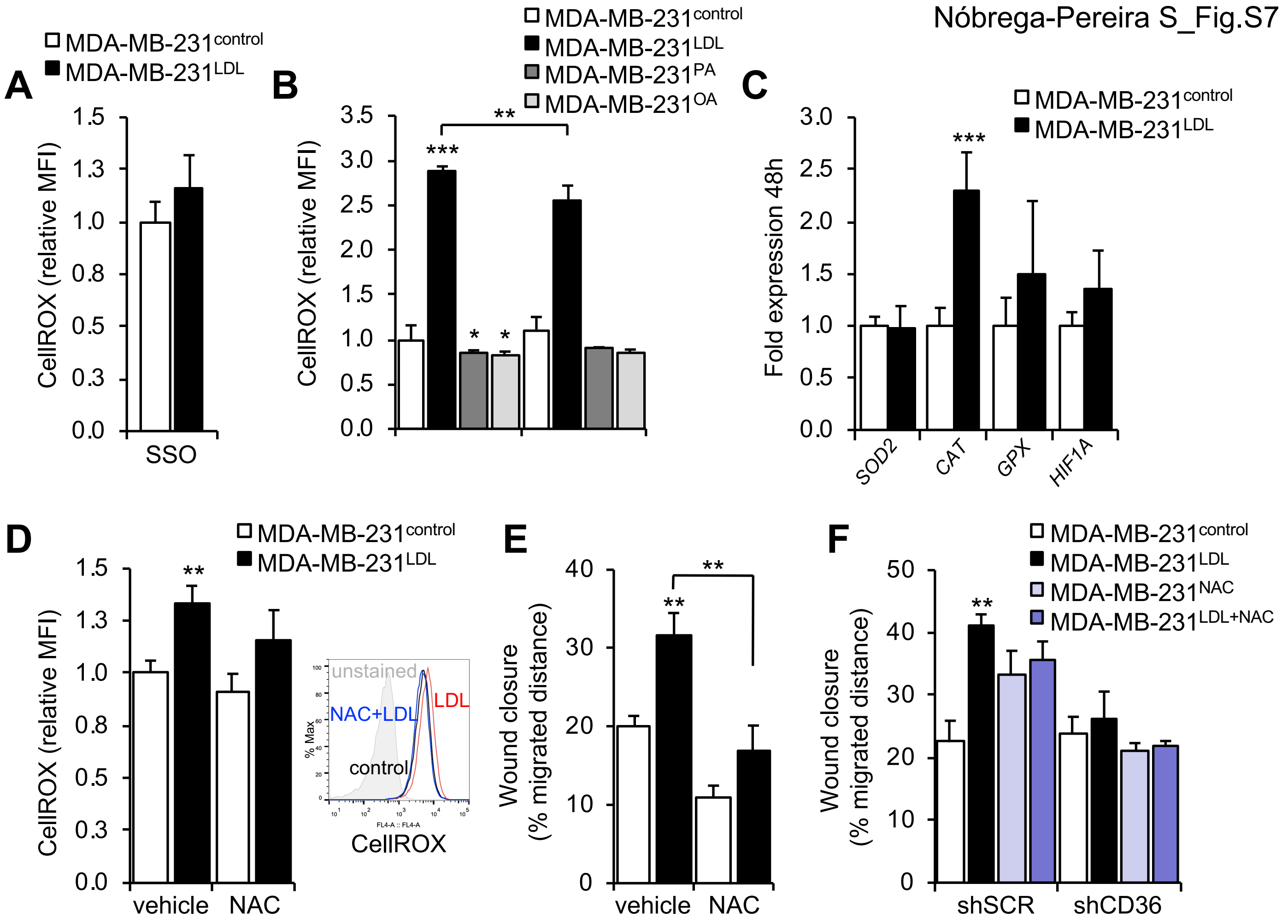

Supplementary Figure S7. Reactive oxygen species formation downstream of mitochondria fatty acid entry is required for LDL-induced migration of breast cancer cells. (A) Flow cytometry quantification of CellROX Deep Red staining depicted as relative median fluorescence intensity (MFI) in control or LDL-exposed MDA-MB-231 cells in the presence of SSO (75 μmol/L, n=6 each). (**B)** Flow cytometry quantification of CellROX Deep Red staining depicted as relative median fluorescence intensity (MFI) in control, LDL, palmitic acid (PA, 50 μmol/L) or oleic acid (OA, 50 μmol/L) exposed MDA-MB-231 cells (n=4/7 each). **(C)** qPCR analysis of the relative expression of the indicated genes in untreated (control) or LDL-exposed MDA-MB-231 cells (n=4/5 each). (**D-E)** Flow cytometry quantification of CellROX Deep Red staining depicted as relative median fluorescence intensity (MFI, left), representative histograms (right) (**D**) and wound closure (**E**) for control or LDL-exposed MDA-MB-231 cells in the absence (vehicle) or presence of *N*-acetylcysteine (NAC, 5 mmol/L) (n=3 each). (**F)** Wound closure of shSCR and shCD36 control or LDL-exposed MDA-MB-231 cells in the absence or presence of *N*-acetylcysteine (NAC, 5 mmol/L, n=3 each). Data are presented as mean ± s.d. * p<0.05, ** p<0.01, *** p<0.001.
