# Supplementary material for: Mitochondrial Metabolism Drives Low-density Lipoprotein-induced Breast Cancer Cell Migration
Source: Cancer Res Commun. 2023 Apr 26;3(4):709–24. doi: 10.1158/2767-9764.CRC-22-0394 (PMC10132314; doi:10.1158/2767-9764.CRC-22-0394)
Supplement: Supplementary Figure S8 — Uncropped membranes relative to the western blot for Drp1, Mfn1, Mfn2 and b-ACTIN proteins displayed in Fig. 2J [file crc-22-0394-s08.pdf]

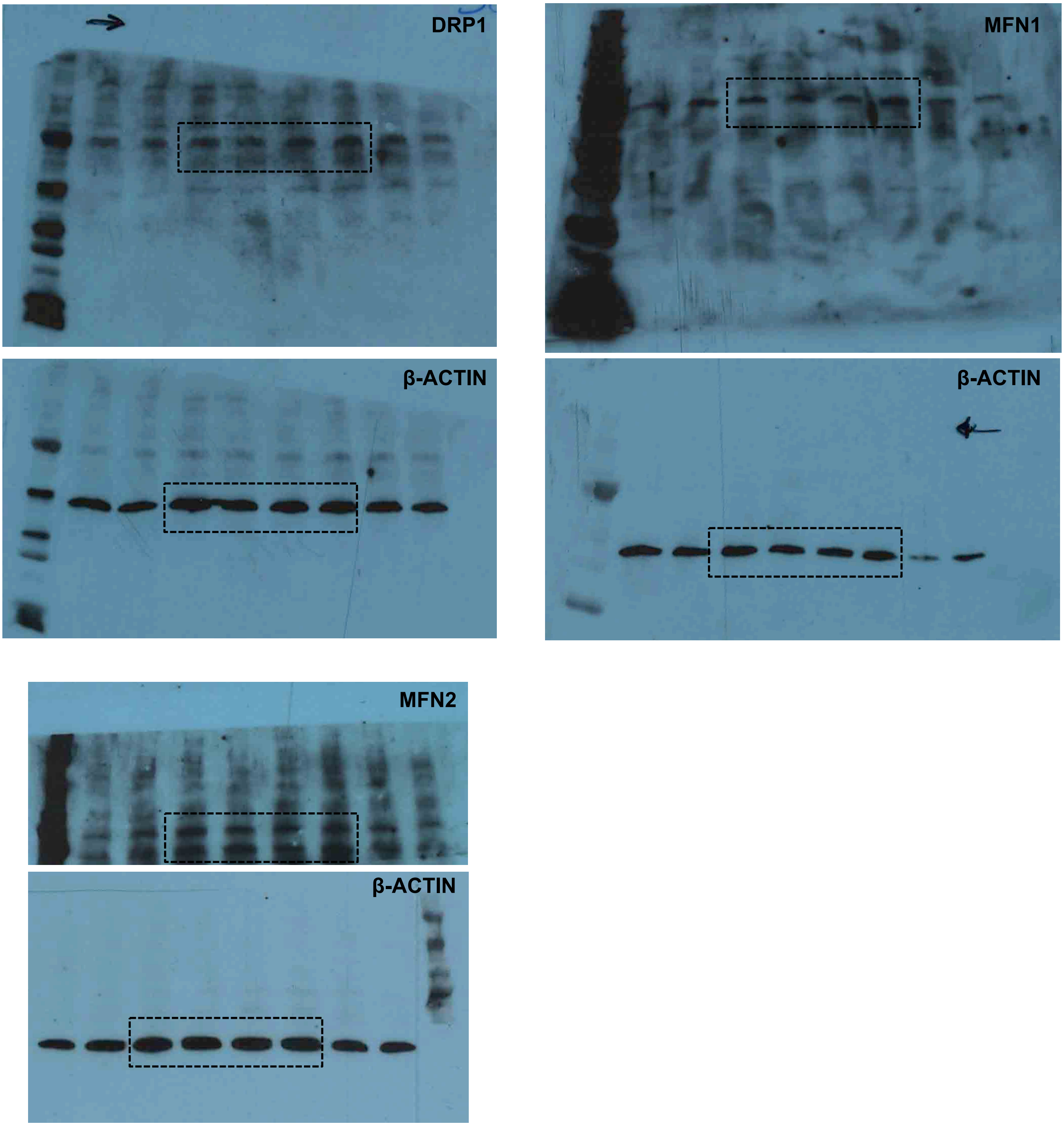

Supplementary Figure S8. Uncropped membranes relative to the western blot for DRP1, MFN1, MFN2 and β-ACTIN proteins displayed in Fig. 2J.
