# Supplementary material for: Mitochondrial Metabolism Drives Low-density Lipoprotein-induced Breast Cancer Cell Migration
Source: Cancer Res Commun. 2023 Apr 26;3(4):709–24. doi: 10.1158/2767-9764.CRC-22-0394 (PMC10132314; doi:10.1158/2767-9764.CRC-22-0394)
Supplement: Supplementary Table S1 — List of primers used for measurements of gene expression levels and mtDNA content by quantitative real-time PCR. [file crc-22-0394-s09.docx]

**Table S1** – List of primers used for measurements of gene expression levels and mtDNA content by quantitative real-time PCR.

name sequence

hND1-F 5’-CCCTAAAACCCGCCACATCT-3’

hND1*-*R 5’-GAGCGATGGTGAGAGCTAAGGT-3’

hβ2-microglobulin-F 5’-TCGCTCCGTGGCCTTAGCTGT-3’

hβ2-microglobulin*-*R 5’-CTTTGGAGTACGCTGGATAGCCTCC-3’

h*18S*-F 5’-GCCCTATCAACTTTCGATGGT-3’

h*18S*-R 5’-CCGGAATCGAACCCTGATT-3’

h*LDHA*-F 5’-ACCCAGTTTCCACCATGATT-3’

h*LDHA-*R 5’-CCCAAAATGCAAGGAACACT-3’

h*PFKFB3*-F 5’-ATTGCGGTTTTCGATGCCAC-3’

h*PFKFB3-*R 5’-GCCACAACTGTAGGGTCGT-3’

h*PKM2*-F 5’-CCACTTGCAATTATTTGAGGAA-3’

h*PKM2-*R 5’-GTGAGCAGACCTGCCAGACT-3’

h*FASN*-F 5’-CGACAGCACCAGCTTCGCCA-3’

h*FASN-*R 5’-CACGCTGGCCTGCAGCTTCT-3’

h*HMGCR*-F 5’-CCAAACCCCGTAACCCAAAG-3’

h*HMGCR-*R 5’-AGCGACTATGAGCGTGAACAA-3’

h*CPT1A*-F 5’-ATGCGCTACTCCCTGAAAGTG-3’

h*CPT1A-*R 5’-GTGGCACGACTCATCTTGC-3’

h*ATP5G1*-F 5’-GCTGTTGTACCAGGGGTCTAA-3’

h*ATP5G1-*R 5’-CTGGCGTGGGAAGTTGCTGT-3’

h*COX5B*-F 5’-GCTGCATCTGTGAAGAGGACAAC-3’

h*COX5B-*R 5’-CAGCTTGTAATGGGTTCCACAGT-3’

h*NDUFB5*-F 5’-CTTCCTCACTCGTGGCTTTC-3’

h*NDUFB5-*R 5’-TTTCCCATGGTCTCCACTGT-3’

h*ACADVL*-F 5’-ACGGGCGTACTGGGTGTT-3’

h*ACADVL-*R 5’-ATGGTGGAGGAGACCACTTG-3’

h*PPARGC1A*-F 5’-CACCAGCCAACACTCAGCTA-3’

h*PPARGC1A*-R 5’-GTGTGAGGAGGGTCATCGTT-3’

h*PPARGC1B*-F 5’-GGCAGGCCTCAGATCTAAAA-3’

h*PPARGC1B*-R 5’-TCATGGGAGCCTTCTTGTCT-3’

h*NRF1*-F 5’-CCATCTGGTGGCCTGAAG-3’

h*NRF1*-R 5’-GTAGTGCCTGGGTCCATGA-3’

h*ESRRA*-F 5’-GGCGGCAGAAGTACAAGC-3’

h*ESRRA*-R 5’-ATTCACTGGGGCTGCTGT-3’

h*TFAM*-F 5’-GAACAACTACCCATATTTAAAGCTCA-3’

h*TFAM*-R 5’-GAATCAGGAAGTTCCCTCCA-3’

h*PPARA*-F 5’-AGAGTGGGCTTTCCGTGTC-3’

h*PPARA*-R 5’-GCCGCCTTCAGGTACAGTAG-3’

h*CD36*-F 5’-GGTGTGGTGATGTTTGTTGC-3’

h*CD36*-R 5’-CAGGGCCTAGGATTTGTTGA-3’

h*LDL-R*-F 5’-GCTTGTCTGTCACCTGCAAA-3’

h*LDL-R*-R 5’-AACTGCCGAGAGATGCACTT-3’

h*SREBP1*-F 5’-CTGTGGGTGAGATCATGTGG-3’

h*SREBP1*-R 5’-GCCAGAAGTCAACCTTGCTC-3’

h*SOD2*-F 5’-GCTCCGGTTTTGGGGTATCTG-3’

h*SOD2*-R 5’-GCGTTGATGTGAGGTTCCAG-3’

h*CAT*-F 5’-TGTTGCTGGAGAATCGGGTTC-3’

h*CAT*-R 5’-TCCCAGTTACCATCTTCTGTGTA-3’

h*GPX*-F 5’-CAGTCGGTGTATGCCTTCTCG-3’

h*GPX*-R 5’-GAGGGACGCCACATTCTCG-3’

h*HIF1A*-F 5’-CATAAAGTCTGCAACATGGAAGGT-3’

h*HIF1A*-R 5’-ATTTGATGGGTGAGGAATGGGTT-3’
