# Supplementary material for: Mitochondrial Metabolism Drives Low-density Lipoprotein-induced Breast Cancer Cell Migration
Source: Cancer Res Commun. 2023 Apr 26;3(4):709–24. doi: 10.1158/2767-9764.CRC-22-0394 (PMC10132314; doi:10.1158/2767-9764.CRC-22-0394)
Supplement: Supplementary Table S2 — Fatty acid profile in relative content (%). Qualitative analysis was calculated by dividing each raw area by the sum of total raw areas. Dash (‘-‘) represents lipid species that were not identified in the sample. Legend: CTR (control), ETO (etomoxir, 100 µM). [file crc-22-0394-s10.docx]

**Table S2** – Fatty acid profile in relative content (%). Qualitative analysis was calculated by dividing each raw area by the sum of total raw areas. Dash (‘-‘) represents lipid species that were not identified in the sample. Legend: CTR (control), ETO (etomoxir, 100 μmol/L).
